# Care coordination through primary health care and network integration: the importance of ensuring comprehensive and integrated care in the Brazilian Unified National Health System

**DOI:** 10.1590/0102-311XEN226625

**Published:** 2026-11-02

**Authors:** Patty Fidelis de Almeida, Simone Schenkman, Aylene Bousquat, Elisandréa Sguario Kemper, Ligia Giovanella

**Affiliations:** 1 Instituto de Saúde Coletiva, Universidade Federal Fluminense, Niterói, Brasil.; 2 Faculdade de Saúde Pública, Universidade de São Paulo, São Paulo, Brasil.; 3 Secretaria de Atenção Primária à Saúde, Ministério da Saúde, Brasília, Brasil.; 4 Centro de Estudos Estratégicos da Fiocruz Antonio Ivo de Carvalho, Fundação Oswaldo Cruz, Fiocruz, Rio de Janeiro, Brasil.

**Keywords:** Integration of Health Services, Integrated Delivery of Health Care, Primary Health Care, Integración de los Servicios de Salud, Prestación Integrada de Atención de Salud, Atención Primaria de Salud

## Abstract

Care coordination and the integration of primary health care (PHC) within the health care network are structural challenges to achieving comprehensive care in the Brazilian Unified Health System (SUS). This article analyzes the prevalence of instruments, strategies and mechanisms for care coordination and health service regulation in basic health units (BHU), as well as their association with contextual and structural factors. Results from the 2024 *Brazilian National Basic Health Units Census* (BHU Census) were analyzed, covering 43,254 units with PHC teams, based on the following components: coordination, network integration, health service regulation mechanisms and shared care. Communication between services occurred predominantly through informal means, while the use of email and interoperable electronic health records was more limited. Referrals through the regulation system took place in about two-thirds of BHU, alongside other pathways under the patient’s responsibility. Electronic health records were widely implemented in BHU, but inadequately shared with other health facilities in the network. Care coordination was stronger between BHU, Psychosocial Care Centers and hospitals. The presence of management within BHU was associated with more frequent and formal communication, as well as greater use of coordination instruments. The findings highlight the need to advance structural policies that establish PHC as the coordinator of care, with investments in digital infrastructure, interoperability of information systems, strengthening of managerial capacity and recognition of multiprofessional work, in order to expand service effectiveness and reduce socio-spatial inequalities.

## Resumen

La coordinación del cuidado y la integración de la atención primaria de salud (APS) en la red de atención sanitaria representan desafíos estructurales para la consolidación de la atención integral en el Sistema Único de Salud (SUS) de Brasil. Este artículo analiza la prevalencia del uso de instrumentos, estrategias y mecanismos para la coordinación del cuidado y la regulación asistencial en las unidades básicas de salud (UBS) y su asociación con factores contextuales y estructurales. Se analizaron los resultados del *Censo Nacional de las Unidades Básicas de Salud* (Censo de las UBS) brasileño, en el 2024, relativos a 43.254 unidades con equipos de APS, con base en los siguientes componentes: coordinación, integración en la red, mecanismos de regulación asistencial y cuidado compartido. La comunicación entre servicios se daba principalmente por medios informales. El uso del correo electrónico y de los historiales médicos electrónicos interoperables estaba más restringido. En aproximadamente dos tercios de las UBS, se produjeron derivaciones a través del sistema de regulación, en paralelo con otros flujos bajo la responsabilidad del usuario. El uso de historiales médicos electrónicos estuvo muy extendido en las UBS, pero su intercambio con otros puntos de la red era limitado. Se demostró que la coordinación del cuidado fue mayor entre las UBS, los Centros de Atención Psicosocial y los hospitales. La presencia de gestores en las UBS se asoció con una mayor asistencia, formalización de la comunicación y adopción de herramientas de coordinación. Los resultados señalan la necesidad de fortalecer las políticas estructurales que consoliden la APS como coordinadora del cuidado, con inversiones en infraestructura digital, interoperabilidad de los sistemas de información, cualificación de la función de gestión y valoración del trabajo multiprofesional, con el fin de ampliar la capacidad de resolución de los servicios y reducir las desigualdades socioespaciales.

## Introduction

Health systems face growing challenges, particularly regarding the changes required to ensure the longitudinal care of people living with noncommunicable diseases and multimorbidity ^1^, in a way that is sustainable over the long term ^2^. Additionally, they are under continuous pressure to incorporate biomedical technologies that diversify providers and may contribute to greater fragmentation of care, thereby increasing the need for coordination and integration among services, professionals, levels of care and other sectors such as social assistance and community services.

Although evidence-based programs for the management of noncommunicable diseases have advanced - especially through the provision of multiprofessional care - the emphasis on specific conditions limits the capacity to deliver comprehensive, patient- and family-centered responses ^3^. In health systems guided by primary health care (PHC), teams are expected to coordinate care across the continuum of services ^4^. When PHC assumes this coordinating role, patients receive higher-quality care, reflected in individualized care plans, encouragement of active participation in decision-making and self-management, and greater satisfaction ^3^. Even so, poor coordination across levels of care is associated with contradictory health guidance, overprescription of medications and increased hospitalizations.

It is assumed that policies and actions aimed at strengthening PHC enhance its capacity to coordinate care ^5^. A robust PHC is essential to improve the efficiency and quality of health systems by promoting continuity of care; expanding equity and access, especially for vulnerable populations; and enhancing system resilience in the face of health crises and climate emergencies ^6^. At the same time, it is essential to develop and systematically implement strategies and instruments that enable coordination ^7^, as well as to address issues related to access to and organization of specialized care ^8^.

Within the Brazilian Unified National Health System (SUS, acronym in Portuguese), care coordination is primarily assigned to Family Health Strategy (FHS) teams ^5^. In this article, coordination is understood as an intentional process aimed at establishing connections among services, actions and health professionals involved in providing care to patients, families and caregivers, ensuring quality, high value, timeliness, continuity and comprehensiveness ^9^. It is argued that the capacity of PHC to coordinate care is conditioned, among other factors, by policies, practices and mechanisms that enable integration across levels of care, in close relation to health service regulation processes ^9^.

Structured mechanisms for communication and information sharing - which reduce duplication, improve continuity of care, support safer clinical decisions and integrate different services, especially for people with complex chronic conditions - are important tools for care coordination ^10^. Health service regulation, in turn, seeks to ensure that all health resources are distributed in a fair, accessible and timely manner ^11^. Thus, interoperable information systems, clinical protocols, telehealth and clearly defined care pathways within Health Care Networks (RAS, acronym in Portuguese), combined with clinical micro-regulation strategies implemented by PHC teams, are essential mechanisms to enable care coordination and improve the quality and appropriateness of referrals to other health facilities in the health system ^11^.

Based on the results of the 2024 *Brazilian National Basic Health Units Census* (BHU Census) ^12^, this article analyzes the prevalence of instruments, strategies and mechanisms for care coordination and health service regulation in Brazilian basic health units (BHU), as well as their association with contextual factors (regions, urban-rural status of municipalities, population size) and structural factors (presence of a manager, presence of a physician with residency training or specialist certification in Family and Community Medicine - FCM -, and the number of family health teams - FHT - within the BHU). Drawing on a broad diagnostic derived from the BHU Census results, this study aims to provide evidence to inform public policies, service organization and health practices aimed at strengthening care coordination and the integration of PHC within the health care network.

## Methodology

The article analyzes the results of the 2024 BHU Census. The data collection instrument was developed based on a set of essential PHC dimensions, understood as comprehensive, effective, community-based, territorial and integrated with the health care network, in line with the FHS care model as described in Bousquat et al. ^12^.

The dimensions of care coordination, integration with the health care network and shared care - present in the instrument - were examined ^13^. The following variables were selected to analyze the prevalence of instruments, strategies and mechanisms for care coordination and health service regulation: (1) instruments and frequency of communication between the BHU and other health facilities in the network; (2) mechanisms for care coordination and the sharing of electronic health records; (3) services with which BHU professionals share care; (4) modes of referral for patients seen at the BHU to specialized consultations; (5) instruments and strategies for health service regulation in the BHU.

According to the adopted framework, to perform the role of care coordinator, PHC must use specific instruments and mechanisms that enable interprofessional communication and the sharing of clinical information, combined with strategies and processes that facilitate access to therapeutic support ^9^ ^,^ ^14^. At the same time, although centralized systems for scheduling specialized procedures are available in nearly all Brazilian municipalities ^15^, clinical micro-regulation processes are strategic for improving the quality of referrals made by PHC teams, based on priority criteria and the monitoring of waiting lists and times, thereby ensuring equitable access to specialized and hospital services ^11^.

Data were collected between June and September 2024 through an electronic procedure conducted in the *e-Gestor Atenção Primária à Saúde* system (https://egestoraps.saude.gov.br/). Initially, all 49,738 facilities registered as PHC services in the Brazilian National Registry of Health Establishments (CNES, acronym in Portuguese), as of April 2024 were included in the BHU Census database. Of this total, 4,026 facilities were deactivated by municipal managers because they were not operating during the response period or did not qualify as PHC services, resulting in 45,712 responding facilities.

Following the assessment of database consistency and completeness, data from 44,938 establishments classified as BHU were analyzed. This total also includes support points linked to BHU that were registered in the CNES but did not have a regular team presence.

Considering that, in the absence of teams, coordination and integration with the network are not feasible, establishments without at least one FHT, primary care teams (PHCT) and/or ffamily health teams for riverine populations (RFHT) were excluded from this analysis. This resulted in a final sample of 43,254 BHU, distributed across the Brazilian regions as follows: North = 3,722; Northeast = 7,164; Southeast = 12,956; South = 6,364; Central-West = 3,048.

To assess associations with macro-level factors, the following contextual variables were considered: (a) Brazilian regions; (b) urban-rural status; and (c) municipality population size.

Urban-rural status was based on the Brazilian Institute of Geography and Statistics (IBGE, acronym in Portuguese) typology ^16^. Municipalities classified as adjacent rural, remote rural and remote intermediate were grouped as rural and/or remote, accounting for 61.5% of municipalities and 15,451 BHU (35.7%). Urban and adjacent intermediate municipalities accounted for 38.5% of municipalities and 27,803 BHU (64.3%).

Population size was defined according to IBGE classification: small I (up to 20,000 inhabitants); small II (20,001 to 50,000 inhabitants); medium (50,001 to 100,000 inhabitants); large (100,001 to 900,000 inhabitants); and metropolitan areas (more than 900,000 inhabitants).

The following structural variables were considered: (a) presence of a manager in the BHU, either in an exclusive role or combined with care provision (34,005 BHU; 78.6%); (b) presence of a physician with residency training or specialist certification in FCM (12,855 BHU; 29.7%); (c) number of FHT in the BHU (distributed as follows: 1 FHT: 30,042 [69.5%]; 2 FHT: 4,998 [11.6%]; 3 FHT: 1,974 [4.6%]; 4 FHT or more: 2,712 [6.3%]).

To examine the role and relative contribution of each structural and contextual factor in the outcomes, bivariate and multivariate analyses were conducted using multilevel logistic regression. The municipal context was incorporated for variables related to referral modes for specialized consultations - with appointments scheduled and communicated to the patient at the time of the visit or later by the BHU - as well as for variables related to health service regulation instruments and strategies (technical protocols, waiting lists and patient tracking, nurse-led referrals, presence of a professional responsible for regulation and the monitoring of patient waiting times). All variables were mutually adjusted.

Odds ratios (OR), 95% confidence intervals (95%CI) and p-values are presented, along with the municipal-level intercept and the intraclass correlation coefficient, highlighting the importance of multilevel analysis ^17^ in capturing the effects of local and regional variables on referral modes and health service regulation strategies. The analyses were performed using Stata software, version 14.2 (https://www.stata.com).

The BHU Census study was approved by the Research Ethics Committee of the School of Public Health, University of São Paulo (CAAE 78767024.2.0000.5421 and approval number 6,779,222).

## Results

The main instruments used for communication between professionals in BHU and other facilities in the network were WhatsApp messages (66.7%) - a text and audio messaging application specified in the BHU Census instrument - and telephone contact (62.8%), while communication via email was less frequent (33.5%). The combined use of all three instruments was reported by 26.8% of BHU, whereas 21.2% did not use any of these means. Although most BHU used at least one instrument, communication was frequent in fewer than half of them (41%). The South and Southeast regions showed greater diversity and frequency in the use of these instruments, while the North and Northeast regions had the lowest. The Central-West region showed intermediate results, closer to the national pattern (Table 1).

**Table 1:** Instruments and frequency of communication between basic health units (BHU) professionals and those at other levels of care in the network, by major regions *, Brazil, rural and urban municipalities, and presence of a manager **, 2024.

Instruments/Frequence	North	Northeast	Southeast	South	Central-West	Brazil	Rural an/or remote	Urban and adjacent intermediate areas	No manager	Manager
	n (%)	n (%)	n (%)	n (%)	n (%)	n (%)	n (%)	n (%)	n (%)	n (%)
WhatsApp	2,151 (57.8)	10,386 (60.5)	9,233 (71.3)	4,968 (78.1)	2,123 (69.7)	28,861 (66.7)	9,985 (64.6)	18,876 (67.9)	6,226 (67.5)	22,625 (66.5)
Phone	1,747 (46.9)	8,449 (49.2)	9,935 (76.7)	5,049 (79.3)	1,963 (64.4)	27,143 (62.8)	8,381 (54.2)	18,762 (67.5)	5,507 (59.7)	21,629 (63.6)
Email	547 (14.7)	2,686 (15.7)	6,883 (53.1)	3,505 (55.1)	846 (27.8)	14,467 (33.5)	3,039 (19.7)	11,428 (41.1)	2,496 (27.0)	11,965 (35.2)
All three	439 (11.8)	1,993 (11.6)	5,545 (42.8)	2,935 (46.1)	667 (21.9)	11,579 (26.8)	2,430 (15.7)	9,149 (32.9)	2,043 (22.1)	9,532 (28.0)
None	1,233 (33.1)	5,060 (29.5)	1,678 (12.9)	620 (9.7)	563 (18.5)	9,154 (21.2)	3,954 (25.6)	5,200 (18.7)	2,007 (21.8)	7,140 (21.0)
Communication frequence (always/almost always)	1,601 (43.0)	6,444 (37.6)	5,988 (46.2)	2,721 (42.8)	1,254 (41.1)	18,008 (41.6)	6,317 (40.9)	11,691 (42.1)	3,022 (32.7)	14,979 (44.1)
Total BHU by region/context	3,722 (8.6)	17,164 (39.7)	12,956 (29.9)	6,364 (14.7)	3,048 (7.1)	43,254 (100.0)	15,451 (35.7)	27,803 (64.3)	9,231 (21.3)	34,005 (78.6)

Source: BHU Census ^12^.

* Number of responses of BHU with teams: Brazil = 43,254; North = 3,722; Northeast = 17,164; South = 12,956; Southeast = 6,364; Central-West = 3,048; (missing = 2);

** Manager (missing = 18).

Inequalities in the use of communication instruments were also observed between urban and rural municipalities. Urban areas reported greater use of telephone (67.5%) and email (41.1%), while in rural municipalities only 15.7% reported using all three communication tools. The presence of a manager in the BHU was associated with greater use of telephone and email, as well as the combined use of all three instruments and higher frequency of communication (“always/almost always”). Notably, between 10% and 30% of BHU, depending on the region, did not use any of the three instruments, with lower usage rates in BHU located in the North/Northeast regions, in rural contexts and in those without a manager (Table 1).

The BHU Census results revealed high use of electronic health records in Brazilian PHC (88.7% of BHU). However, the sharing of records with other levels of care in the health network - for communication and care coordination purposes - occurred in only 50.8% of BHU, with significant regional differences (South: 73.5%; North: 31.3%). It should be noted that record sharing occurred primarily with other BHU (70.9%). For continuity of care with other services in the Health Care Network, however, this proportion was below one quarter of establishments, with varying percentages depending on the type of service: Psychosocial Care Centers (CAPS, acronym in Portuguese) (21.1%), public specialized services (18.8%), emergency services/Urgent Care Units (UPA, acronym in Portuguese) (18.4%) and Specialized Dental Centers (CEO, acronym in Portuguese) (17.7%). Even lower percentages were found for laboratories and public hospitals (Table 2).

**Table 2:** Care coordination strategies and sharing of electronic health records across levels of care in basic health units (BHU), by major regions *, Brazil, rural and urban municipalities, and presence of a manager **, 2024.

	North	Northeast	Southeast	South	Central-West	Brazil	Rural an/or remote	Urban and adjacent intermediate areas	No manager	Manager
	n (%)	n (%)	n (%)	n (%)	n (%)	n (%)	n (%)	n (%)	n (%)	n (%)
Coordination strategies										
Technical meetings or joint clinical sessions	1,766 (47.5)	8,037 (46.8)	7,994 (61.7)	3,562 (56.0)	1,317 (43.2)	22,676 (52.4)	7,178 (46.5)	15,498 (55.7)	4,261 (46.2)	18,407 (54.1)
Shared consultations	787 (21.1)	5,107 (29.8)	4,029 (31.1)	1,782 (28.0)	768 (25.2)	12,473 (28.8)	3,922 (25.4)	8,551 (30.8)	2,235 (24.2)	10,234 (30.1)
Shared care plan/PTS	400 (10.8)	3,255 (19.0)	1,097 (8.5)	1,919 (30.2)	396 (13.0)	7,041 (16.3)	1,874 (12.1)	5,624 (20.2)	1,303 (14.1)	6,194 (18.2)
Telehealth	407 (9.9)	3,317 (18.7)	1,120 (8.4)	1,947 (29.5)	370 (12.1)	7,163 (15.9)	2,493 (16.1)	4,548 (16.4)	1,202 (13.0)	5,839 (17.2)
Referral and counter-referral form	1,876 (50.4)	8.512 (49.6)	8,354 (64.5)	3,611 (56.7)	1,596 (52.4)	23,949 (55.4)	7,106 (46.0)	16,843 (60.6)	4,697 (50.9)	19,246 (56.6)
Receives hospital discharge summaries (always/almost always)	760 (20.4)	3,714 (21.6)	5,454 (42.1)	1,732 (27.2)	584 (19.2)	12,244 (28.3)	3,698 (23.9)	8,546 (30.7)	2,028 (22.0)	10,211 (30.0)
PEC communication with professionals at other health facilities fof the network	1,164 (31.3)	6,842 (39.9)	7,521 (58.1)	4,680 (73.5)	1,773 (58.2)	21,980 (50.8)	6,294 (40.7)	15,686 (56.4)	4,197 (45.5)	17,782 (52.3)
Services with which BHU share PEC										
Other BHU	1,694 (45.5)	11,105 (64.7)	9,876 (76.2)	5,457 (85.8)	2,541 (83.4)	30,673 (70.9)	9,506 (61.5)	21,167 (76.1)	6,384 (69.2)	24,280 (71.4)
CAPS	282 (7.6)	1,257 (7.3)	3,716 (28.7)	3,246 (51.0)	635 (20.8)	9,136 (21.1)	914 (5.9)	8,222 (29.6)	1,541 (16.7)	7,592 (22.3)
Public specialized services	205 (5.5)	1,357 (7.9)	3,706 (28.6)	2,270 (35.7)	599 (19.7)	8,137 (18.8)	1,019 (6.6)	7,118 (25.6)	1,356 (14.7)	6,777 (19.9)
Emergency departments and other urgent care services/UPA	188 (5.0)	834 (4.9)	3,367 (26.0)	2,765 (43.5)	810 (26.6)	7,964 (18.4)	1,170 (7.6)	6,794 (24.4)	1,316 (14.3)	6,645 (19.5)
CEO	316 (8.5)	1,768 (10.3)	3,084 (23.8)	1,976 (31.0)	502 (16.5)	7,646 (17.7)	592 (3.8)	7,054 (25.4)	1,207 (13.1)	6,437 (18.9)
Diagnostic and laboratory services	215 (5.8)	896 (5.2)	2,772 (21.4)	1,718 (27.0)	696 (22.8)	6,297 (14.6)	939 (6.1)	5,358 (19.3)	989 (10.7)	5,307 (15.6)
Public hospitals	125 (3.4)	615 (3.6)	1,234 (9.5)	597 (9.4)	423 (13.9)	2,994 (6.9)	606 (3.9)	2,388 (8.6)	449 (4.9)	2,543 (7.5)
Total BHU by region/context	3,722 (8.6)	17,164 (39.7)	12,956 (29.9)	6,364 (14.7)	3,048 (7.1)	43,254 (100.0)	15,451 (35.7)	27,803 (64.3)	9,231 (21.3)	34,005 (78.6)

CAPS: Psychosocial Care Centers; CEO: Specialized Dental Centers; PEC: Citizen’s Electronic Health Record; PTS: Individualized Therapeutic Plans; UPA: Urgent Care Units.

Source: BHU Census ^12^.

* Number of responses of BHU with teams: Brazil = 43,254; North = 3,722; Northeast = 17,164; South = 12,956; Southeast = 6,364; Central-West = 3,048; (missing = 2);

** Manager (missing = 18).

Among the coordination strategies and mechanisms examined in Table 2, joint technical meetings/clinical sessions (52.4%) and referral/counter-referral forms (55.4%) were the most well established. However, the use of Individualized Therapeutic Plans (PTS, acronym in Portuguese), telehealth (teleconsulting), shared consultations and the regular receipt of hospital discharge summaries remained infrequent. The best performances were observed in the Southeast region for joint clinical sessions (61.7%) and receipt of hospital discharge summaries (42.1%), and in the South region for the use of PTS and telehealth (around 30%). The absence of any coordination mechanism reached nearly 10% of BHU, with the worst results in the North region. Urban municipalities and BHU with a manager showed greater adoption of coordination mechanisms and stronger integration via electronic health records with services across the RAS (Table 2).

The BHU Census results also showed that the services with which BHU professionals most frequently shared care were CAPS (74.6%) and hospitals (67%). In around 50% of BHU, links were established with maternity services, CEO, outpatient specialty services and services for sexually transmitted infections (STIs). Less frequent coordination was observed with rehabilitation services and polyclinics. Strong regional inequalities were evident, with the Southeast Region showing greater integration for nearly all listed services, while the North and Northeast regions showed the lowest levels, except for polyclinics (which were more common in the Northeast Region). Rural-urban differences were also pronounced, especially in relation to mental health services, CEO, outpatient care, STI services and rehabilitation. The presence of a manager was consistently associated with higher levels of shared care across all specialized services (Table 3).

**Table 3:** Services with which basic health units (BHU) professionals share care, by major regions *, Brazil, rural and urban municipalities, and presence of a manager **, 2024.

Service	North	Northeast	Southeast	South	Central-West	Brazil	Rural an/or remote	Urban and adjacent intermediate areas	No manager	Manager
	n (%)	n (%)	n (%)	n (%)	n (%)	n (%)	n (%)	n (%)	n (%)	n (%)
CAPS	2,451 (65.8)	12,473 (72.7)	10,721 (82.7)	4,635 (72.8)	1,975 (64.8)	32,255 (74.6)	8,588 (55.6)	23,667 (85.1)	6,597 (71.5)	25,645 (75.4)
Hospitals	2,453 (65.9)	11,950 (69.6)	8,468 (65.4)	4,145 (65.1)	1,986 (65.2)	29,002 (67.0)	10,586 (68.5)	18,416 (66.2)	5,863 (63.5)	23,129 (68.0)
Maternities	1,701 (45.7)	9,032 (52.6)	7,536 (58.2)	3,441 (54.1)	1,121 (36.8)	22,831 (52.8)	6,634 (42.9)	16,197 (58.3)	4,231 (45.8)	18,595 (54.7)
CEO	1,296 (34.8)	8,432 (49.1)	7,547 (58.3)	3,211 (50.5)	1,343 (44.1)	21,829 (50.5)	4,964 (32.1)	16,865 (60.7)	4,089 (44.3)	17,737 (52.2)
Speciality outpatient center	1,122 (30.1)	7,145 (41.6)	8,085 (62.4)	3,511 (55.2)	1,337 (43.9)	21,200 (49.0)	5,383 (34.8)	15,817 (56.9)	4,049 (43.9)	17,145 (50.4)
Specialized STI care services	2,083 (56.0)	6,869 (40.0)	7,886 (60.9)	3,744 (58.8)	1,510 (49.6)	22,092 (51.1)	4,796 (31.0)	17,296 (62.2)	3,981 (43.1)	18,102 (53.2)
Regional polyclinics	925 (24.9)	7,483 (43.6)	3,897 (30.1)	1,355 (21.3)	699 (22.9)	14,359 (33.2)	4,998 (32.3)	9,361 (33.7)	2,853 (30.9)	11,503 (33.8)
Specialized rehabilitation centers	706 (19.0)	4,067 (23.7)	4,532 (35.0)	1,609 (25.3)	789 (25.9)	11,703 (27.1)	2,906 (18.8)	8,797 (31.6)	1,934 (20.9)	9,769 (28.7)
Total BHU by region/context	3,722 (8.6)	17,164 (39.7)	12,956 (29.9)	6,364 (14.7)	3,048 (7.1)	43,254 (100.0)	15,451 (35.7)	27,803 (64.3)	9,231 (21.3)	34,005 (78.6)

CAPS: Psychosocial Care Centers; CEO: Specialized Dental Centers; STI: sexually transmitted infections.

Source: BHU Census ^12^.

* Number of responses of BHU with teams: Brazil = 43,254; North = 3,722; Northeast = 17,164; South = 12,956; Southeast = 6,364; Central-West = 3,048; (missing = 2);

** Manager (missing = 18).

Multiple modes of referring patients to specialized consultations coexist across Brazilian BHU. The most well established was scheduling through the regulation system, with the appointment date communicated either later (59.6%) or at the time of the visit (27.9%), predominating in the Southeast and South regions and in urban areas. Modes that place responsibility on the patient to seek specialized care - such as receiving a referral form and being instructed to look for a service indicated by the team - were also substantial, reaching approximately 50% in the North, Northeast and Central-West regions, as well as in rural municipalities. Similarly, 7% of BHU advised patients to seek specialized consultations on their own. The presence of physicians with specialization or residency training in FCM, the presence of a manager and a higher number of FHT increased the proportion of appointments scheduled by the BHU (Table 4).

**Table 4:** Referral modes for patients seen at basic health units (BHU) to specialized care, by macrorregions, Brazil, rural and urban municipalities, presence of a physician with residency or specialist certification in Family and Community Medicine (FCM), and presence of a manager in the BHU, 2024.

Referral models	Appointment scheduled by the BHU via the regulation system, with the date informed later		Patient receives a referral form and goes tothe service indicated by the team		Appointment schedule by the patient throught the scheduling/regulation center		Appointment scheduled by the BHU and communicated to the patient at the time of the visit		Patient seeks a specialized consultation on their own		Appointment scheduled by the BHU and communicated to the patient either at the time of the visit or later **	
	n	%	n	%	n	%	n	%	n	%	n	%
Macrorregions												
North	2,037	54.7	1,918	51.5	1,150	30.9	802	21.6	262	7.0	2,342	62.9
Northeast	7,999	46.6	8,321	48.5	8,542	49.8	4,470	26.0	1,522	8.9	9,915	57.8
Southeast	9,565	73.8	4,909	37.9	3,077	23.8	4,287	33.1	735	5.7	10,629	82.0
South	4,531	71.2	2,449	38.5	1,684	26.5	1,872	29.4	305	4.8	4,943	77.7
Central-West	1,628	53.4	1,589	52.1	1,057	34.7	656	21.5	156	5.1	1,850	60.7
Brazil	25,760	59.6	19,186	44.4	15,510	35.9	12,087	27.9	2,980	6.9	29,679	68.6
Rural-urban status												
Rural ans/or remote	6,476	41.8	8,176	52.9	7,037	45.5	3,697	23.9	1,378	8.9	8,087	52.3
Urban and adjacent intermediate areas	19,284	69.4	11,010	39.6	8,473	30.5	8,390	30.2	1,602	5.8	21,592	77.7
Physician with residence or specialist certification in FCM ***												
No	17,133	56.4	13,946	45.9	11,501	37.9	8,196	27.0	2,191	7.2	20,024	65.9
Yes	8,620	67.1	5,235	40.7	4,006	31.2	3,890	30.3	788	6.1	9,647	75.0
Presence of manager												
No	4,767	51.6	4,111	44.5	3,942	42.7	2,295	24.9	701	7.6	5,700	61.8
Yes	20,982	61.7	15,069	44.3	11,565	34.0	9,787	28.8	2,279	6.7	23,967	70.5
Population size												
Large size/Metropolis	10,032	85.7	3,712	31.7	2,246	19.2	3,760	32.1	553	4.7	10,585	90.4
Medium size/Small size	15,728	49.9	15,474	49.1	13,264	42.0	8,327	26.4	2,427	7.7	19,094	60.5
Numberof FHT ^#^												
1	16,308	54.3	14,009	46.6	11,971	39.9	7,945	26.5	2,237	7.4	19,245	64.1
2	3,463	69.3	2,090	41.8	1,485	29.7	1,396	27.9	280	5.6	3,834	76.7
3	1,571	79.6	686	34.8	437	22.1	639	32.4	101	5.1	1,682	85.2
4 or more	2,105	77.6	1,009	37.2	617	22.7	1,009	37.2	135	5.0	2,295	84.6
Appointment scheduled by the BHU and communicated to the patient at the time of the visit or later	Bivariate analysis						Multivariate analysis					
	OR			95%CI			OR			95%CI		
Large size/Metropolis	6.12			5.73-6.53			8.22			6.15-11.00		
Southeast macrorregion	2.70			2.56-2.84			2.86			2.44-3.36		
Urban and adjacent intermediate areas context	3.17			3.03-3.30			2.03			1.74-2.36		
Presence of manager at BHU	1.48			1.41-1.55			1.31			1.19-1.43		
Physiciam with residence or specialist in FCM	1.56			1.48-1.63			1.11			1.03-1.20		
Number of FHT	1.36			1.33-1.39			1.05			1.01-1.10		
Intercept	-			-			0.88			0.77-1.00		
Municipal context	-			-			5.22			4.83-5.64		
ICC	-			-			0.61			0.59-0.63		
LR												
n	43,186-43,252						43,163					
p	< 0.0001						< 0.0001					

95%CI: 95% confidence interval; ICC: intraclass correlation coefficient; FHT: family health teams; LR: likelihood ratio; OR: odds ratio.

Source: BHU Census ^12^.

* Number of responses with teams: Brazil = 43,254; North = 3,722; Northeast = 17,164; Southeast = 12,956; South = 6,364; Central-West = 3,048; (missing = 2);

** Appointments made exclusively in these modes 39.7% of Brazilian BHU (17,179);

*** Physicians with FCM specialization (missing = 17);

^#^ BHU according to number of FHT (missing = 66).

A clear gradient was observed in the scheduling of appointments by BHU through the regulation system, with higher odds of occurrence, even after adjustments in the multivariate model, in municipalities with large populations or metropolitan status (8.22 times); in the Southeast Region (2.86 times); in urban contexts (2.03 times); in units with a manager (1.31 times); with a physician specialized in FCM (1.11 times); and with a greater number of FHT (1.08 times). The municipal context proved highly relevant for patient-centered scheduling modes, with an OR of 5.22 and an intraclass correlation coefficient (ICC) of 61% (Table 4).

The most prevalent health service regulation instruments in BHU were technical protocols for access to specialized care (67.8%) and records/lists of referred patients (57.7%), with better performance in the Southeast Region and in municipalities with more than 100,000 inhabitants (large or metropolitan areas). Nurse-led referrals were present in approximately half of BHU. Strategies such as monitoring waiting times (34.9%) and assigning a professional responsible for regulation within the BHU (35.2%) were less frequent. Notably, all these mechanisms were more common in urban municipalities, in BHU with a manager and in those with a higher number of FHT, followed by services with a physician holding residency training or specialist certification in FCM.

A clear gradient was observed for regulation instruments and strategies, even after multivariate adjustment, with higher odds of occurrence in municipalities with large populations or metropolitan status, particularly for nurse-led referrals and the use of protocols (2.34-3.10 times). Regional differences were also evident: the Southeast Region showed higher odds for waiting time monitoring and patient registries/lists (1.91-1.43 times); the South Region for technical protocols (1.97 times); the Northeast Region for nurse-led referrals (2.06 times); and the North Region for the presence of a professional responsible for regulation (1.28 times).

An association with urban status was also observed for nurse-led referrals, patient lists and especially the use of protocols (1.15-1.88 times). Conversely, after adjustment, urban status was no longer significant for the presence of a professional responsible for regulation or for monitoring patient waiting times. The municipal context remained particularly relevant for nurse-led referral strategies, with an OR of 3.54 and an ICC of 52%, most prevalent in the Northeast Region. The other strategies showed ORs ranging from 2.00-2.46, with ICC values between 38-43%.

The presence of a manager in the BHU was significant, especially for monitoring patient waiting times and for the presence of a professional responsible for regulation (1.81-2.14 times). The presence of at least one physician specialized in FCM (1.15-1.26 times) and a higher number of FHT (1.04-1.10 times) were both more strongly associated with the use of technical protocols and with the existence of a professional responsible for regulation (Table 5).

**Table 5:** Care regulation instruments and strategies in basic health units (BHU), by macrorregions, Brazil, rural and urban municipalities, presence of a physician with residency or specialist certification in Family and Community Medicine (FCM), and presence of a manager, 2024.

Regulation strategies	Technical protocols for access to specialized care services		Record/List of patients referred to other levels of care within the network		Nurse-led referrals		Professional responsible for care regulation in BHU		Monitoring waisting times for patients referred to other levels of care in the network	
	n	%	n	%	n	%	n	%	n	%
Macrorregions										
North	2,423	65.1	2,030	54.5	1,822	48.9	1,566	42.1	1,215	32.6
Northeast	9,853	57.4	8,899	51.9	9,697	56.5	5,223	30.4	5,205	30.3
Southeast	10,046	77.5	8,877	68.5	6,417	49.5	5,346	41.3	5,582	43.1
South	4,934	77.5	3,748	58.9	3,432	53.9	2,140	33.6	2,327	36.6
Central-West	2,090	68.6	1,410	46.3	1,279	42.0	968	31.8	785	25.8
Brazil	29,346	67.8	24,964	57.7	22,646	52.4	15,243	35.2	15,114	34.9
Presence of manager										
No	5,123	55.5	4,477	48.5	4,152	45.0	2,172	23.5	2,280	24.7
Yes	24,211	71.2	20,476	60.2	17,484	54.4	13,063	38.4	12,826	37.7
Physician with residence or specialist certification in FCM **										
No	19,760	65.0	16,987	55.9	15,306	50.4	10,087	33.2	9,939	32.7
Yes	9,576	74.5	7,969	62.0	7,335	57.1	5,153	40.1	5,169	40.2
Rural-urban status										
Rural ans/or remote	8,525	55.2	7,505	48.6	7,059	45.7	4,745	30.7	4,368	28.3
Urban and adjacent intermediate areas	20,821	74.9	17,459	62.8	15,587	56.1	10,498	37.8	10,746	38.7
Population size										
Large size/Metropolis	9,863	84.2	7,968	68.0	7,553	64.5	5,140	43.9	5,338	46.0
Medium size/Small size	19,483	61.8	16,996	53.9	15,093	47.9	10,103	32.0	9,726	30.8
Number of FHT ***										
1	19,100	63.6	16,631	55.4	15,193	50.6	9,770	32.5	9,679	32.2
2	3,744	74.9	3,024	60.5	2,714	54.3	1,974	39.5	1,874	37.5
3	1,628	82.5	1,301	65.9	1,182	59.9	820	41.5	878	44.5
4 or more	2,320	85.6	1,908	70.4	1,792	66.1	1,404	51.8	1,384	51.0
Bivariate analysis	OR	95%CI	OR	95%CI	OR	95%CI	OR	95%CI	OR	95%CI
Large size/Metropolis	3.30	3.12-3.49	1.82	1.74-1.90	1.98	1.89-2.07	1.66	1.59-1.73	1.91	1.83-2.00
Macrorregion	1.76	1.66-1.88	1.92	1.84-2.01	1.32	1.27-1.37	1.37	1.28-1.47	1.65	1.58-1.72
Presence of manager	1.98	1.89-2.08	1.61	1.53-1.68	1.46	1.39-1.53	2.03	1.92-2.14	1.85	1.75-1.95
Urban and adjacent intermediate areas context	2.42	2.32-2.53	1.79	1.72-1.86	1.52	1.46-1.58	1.37	1.31-1.43	1.60	1.53-1.67
Physician with FCM specialization	1.57	1.50-1.64	1.29	1.23-1.34	1.31	1.26-1.36	1.35	1.29-1.40	1.38	1.33-1.44
Number of FHT	1.36	1.33-1.40	1.18	1.16-1.21	1.20	1.17-1.22	1.23	1.21-1.26	1.23	1.20-1.25
LR										
n	43,185-43,249		43,184-43,248		43,185-43,249		43,185-43,249		43,183-43,247	
p	< 0.0001		< 0.0001		< 0.0001		< 0.0001		< 0.0001	
Multivariate analysis	OR	95%CI	OR	95%CI	OR	95%CI	OR	95%CI	OR	95%CI
Large size/Metropolis	3.10	2.02-2.29	1.49	1.24-1.80	2.34	1.85-2.97	1.51	1.23-1.85	1.73	1.43-2.09
Macrorregion	1.97	1.71-2.26	1.91	1.71-2.13	2.06	1.81-2.34	1.28	1.06-1.56	1.43	1.27-1.60
Presence of manager	1.79	1.66-1.92	1.63	1.52-1.74	1.58	1.46-1.70	2.14	1.97-2.31	1.81	1.67-1.95
Urban and adjacent intermediate areas context	1.88	1.68-2.11	1.40	1.26-1.56	1.15	1.01-1.31	0.94	0.84-1.05	1.11	0.99-1.24
Physician with FCM specialization	1.24	1.17-1.32	1.17	1.10-1.23	1.15	1.09-1.22	1.26	1.19-1.33	1.19	1.12-1.26
Number of FHT	1.10	1.06-1.15	1.05	1.01-1.08	1.04	1.01-1.07	1.09	1.06-1.13	1.04	1.01-1.07
Intercept	0.72	0.65-0.79	0.55	0.50-0.61	0.36	0.32-0.40	0.23	0.22-0.25	0.16	0.15-0.18
Municipal context	2.42	2.23-2.62	2.00	1.85-2.17	3.54	3.29-3.82	2.46	2.27-2.66	2.10	1.93-2.29
ICC	0.42	0.40-0.44	0.38	0.36-0.40	0.52	0.50-0.54	0.43	0.41-0.45	0.39	0.37-0.41
LR										
n	43,163		43,162		43,163		43,163		43,161	
p	< 0.0001		< 0.0001		< 0.0001		< 0.0001		< 0.0001	

95%CI: 95% confidence interval; ICC: intraclass correlation coefficient; FHT: family health teams; LR: likelihood ratio; OR: odds ratio.

Source: BHU Census ^12^.

* Number of responses with teams: Brazil = 43,254; North = 3,722; Northeast = 17,164; South = 12,956; Southeast = 6,364; Central-West = 3,048; (missing = 5-7);

** Physicians with FCM specialization (missing = 17);

*** BHU according to number of FHT (missing = 66).

## Discussion 

Care coordination through PHC and its integration into health care networks are among the main challenges faced by universal public health systems ^6^. Overcoming fragmentation is essential to ensure comprehensive, integrated care capable of responding adequately to demographic and epidemiological changes ^3^. Organizing the health care network around PHC is key to ensuring equitable access according to population needs and upholding the principle of comprehensiveness in health care ^9^.

In Brazilian BHU, WhatsApp and telephone are the most frequently cited instruments for communication between professionals within the RAS, while email, which generates more formal and traceable records, remains marginal in the North, Northeast and Central-West regions. The combined use of all three instruments is limited. The predominance of informal means, together with professional turnover ^18^, may compromise the institutionalization and continuity of interprofessional communication. The use of mobile applications and telephone has expanded significantly for health communication and monitoring, especially during the COVID-19 pandemic ^19^; however, in Brazil, most professionals use personal phones to communicate with patients and other professionals ^20^. According to BHU Census data, only 24.8% of BHU have institutional mobile phones, and although internet access has improved, connection quality remains insufficient in part of the network, particularly in the North and Northeast regions ^21^. There is therefore an urgent need to invest in the provision of institutional mobile devices with data plans as part of professionals’ work tools, in order to support interprofessional communication and continuous patient follow-up.

Regional inequalities in the availability of communication tools are significant. The South and Southeast regions consistently showed better results, in line with other studies indicating that regions with poorer socioeconomic indicators - such as gross domestic product (GDP) per capita and the Municipal Human Development Index (M-HDI), particularly the North and Northeast regions - tend to concentrate BHU infrastructure classified as inadequate, rudimentary or limited ^22^ ^,^ ^23^. Nevertheless, BHU Census findings point to improvements in infrastructure between 2012 and 2024 across all Brazilian regions ^24^. This more favorable scenario probably reflects converging policies aimed at addressing workforce shortages, such as More Doctors Programs (PMM, acronym in Portuguese) ^25^, as well as more recent initiatives like *Informatiza UBS* ^26^ and *SUS Digital* program ^27^, which seek to expand digitalization and diversify communication tools among services, health professionals and the population.

The results confirm the widespread adoption of electronic health records in BHU. In recent years, there has been significant expansion in the implementation of the Citizen’s Electronic Health Record (PEC, acronym in Portuguese) across Brazilian municipalities, which, even without mandatory implementation, has become the primary system of choice ^28^. From a care coordination perspective, the sharing of records with other health facilities in the RAS is reported by about half of BHU, with regional differences. Although promising, these findings should be interpreted with caution. Most clinical information sharing via electronic health records occurs between BHU, while exchange with key services for continuity of care - such as CAPS, specialized care services, emergency/urgent care and CEO - remains limited.

Referral and counter-referral forms, along with joint clinical sessions, are the most well established coordination strategies. Although referral and counter-referral forms have long been available within the SUS, referrals from PHC to other services are far more frequent than the reverse, indicating predominantly one-way communication. Strategies of mutual adjustment - such as direct interaction between professionals from different services through shared clinical sessions - are considered essential for achieving collaborative care and building trust among health professionals ^10^ ^,^ ^29^. BHU Census findings indicate greater use of this strategy compared to studies conducted in other contexts ^30^ ^,^ ^31^.

Essential instruments for managing complex conditions - such as the PTS, telehealth, shared consultations and the regular receipt of hospital discharge summaries - show low uptake. As argued in this article, pro-coordination measures require targeted actions and incentives. A case in point is the discharge summary: in one Brazilian municipality it was institutionalized through a medical residency program in public hospitals, contributing to more frequent receipt of the document in PHC ^30^. However, reliance on patients to carry discharge summaries and referral/counter-referral forms still appears to be the predominant pattern, compromising their timely receipt in PHC for decision-making ^30^ ^,^ ^31^.

BHU Census findings indicate a substantial level of shared care with CAPS and hospitals. Brazil’s psychiatric and health care reforms share principles and proposals for transforming the care model. CAPS and BHU are both locally based services and, as observed by Miliauskas et al. ^32^, there appears to be an association between the simultaneous expansion of these services and reductions in psychiatric hospitalization rates in metropolitan areas. This finding suggests that, beyond increased coverage, shared care strategies are also being implemented.

The presence of a manager in the BHU emerges as a key factor even in more vulnerable contexts: facilities with a manager show greater combined use of WhatsApp, telephone and email, higher communication frequency and greater adoption of clinical coordination mechanisms. These findings reinforce that planning and organizing work processes in PHC are decisive for enabling and improving information exchange with other levels of care. Strengthening this managerial function-with professionals trained in health management, capable of analyzing and using indicators, working in teams and communicating effectively, among other skills ^33^ ^,^ ^34^ - may help mitigate territorial inequalities. The lack of specific training in management roles and the accumulation of managerial and care responsibilities, especially in BHU with a larger number of teams, are critical factors that may compromise performance, particularly in activities requiring coordination with other services in the RAS ^34^. Although this article does not present analyses related to the training of BHU managers (a variable included in the BHU Census instrument), the findings suggest that the professional profile described above aligns with competencies found in public health training, particularly among graduates in Collective Health.

Inequalities between urban and rural areas are critical. In urban municipalities, communication via telephone and email is significantly more prevalent than in rural areas. This difference is also reflected in shared care across the RAS: BHU located in urban municipalities show greater coordination with CAPS, hospitals, maternity services, specialized services and STI services, whereas rural areas face greater difficulties in integrating with mental health, specialized oral health and rehabilitation services, also reflecting challenges in accessing therapeutic support in these areas ^35^.

Referrals through the regulation system occur in about two-thirds of BHU, alongside other pathways for which the patient is responsible. Establishing PHC services as gatekeepers alone is insufficient to ensure coordination or access. It is also necessary to guarantee an adequate public supply of services, organize care networks and strengthen problem-solving capacity in PHC, with clearly defined coordination functions, so that patients do not have to navigate circular pathways caused by bureaucratic barriers in the regulation process ^36^. The common practice of providing a physical referral form shifts responsibility away from the BHU for ensuring continuity of care and creates opportunities for patients to seek services in the private sector, increasing the uptake of “low-cost health insurance” and expanding the public-private mix ^37^. At the same time, entering a request into the regulation system cannot be treated as a mere bureaucratic act marking the end of the BHU’s responsibility ^38^, yet this appears to be common, as only about one-third of facilities maintain lists of referred patients or monitor waiting times.

The acceptance of nurse-led referrals to specialized care in more than half of BHU highlights the growing importance of this professional in PHC. The higher prevalence of nurse-led referrals in the Northeast region may be related to its historical context, including earlier universalization of coverage through the FHS and the role of the Community Health Workers Program ^39^, as well as previous shortages of physicians in teams prior to the implementation of PMM ^24^, Similarly, the implementation of comprehensive protocols guiding nursing practice has become increasingly common, along with the presence of residency programs in family and community nursing within BHU, especially in urban centers ^40^.

The observed associations between a higher prevalence of regulation strategies and the presence of a manager, at least one physician specialized in FCM and a greater number of FHT in the BHU point to the need to expand service infrastructure, technical staff and workforce capacity. Care coordination involves managerial functions ^41^, which require professionals with protected time to perform these roles, as well as adequately structured BHU to improve referrals and monitor continuity of care. Moreover, physicians trained in FCM, well integrated within multiprofessional teams, are recognized as essential for quality, care coordination and the delivery of comprehensive PHC ^42^ ^,^ ^43^.

One limitation of this study is the cross-sectional nature of the BHU Census, which makes it impossible to establish causal relationships. In addition, the data were obtained through self-report by respondents, which may introduce information bias. Likewise, the findings do not allow inferences regarding the quality, intensity or routine use of coordination mechanisms, nor their potential impact on patients’ care pathways, indicating the need for further complementary studies.

## Conclusion 

The findings show that, although important advances have been made, care coordination through PHC in Brazil remains strongly conditioned by structural weaknesses, territorial inequalities and the predominance of informal communication mechanisms. Regional, urban-rural and organizational disparities - especially those related to infrastructure, workforce availability and the presence of management in BHU - reinforce that coordination performance depends on concrete institutional, organizational and managerial capacities operating at the local level.

Overcoming the identified obstacles requires strengthening structural policies that enhance the care coordination role of PHC, with sustained investments in digital infrastructure, high-quality connectivity and institutional mobile devices with data plans. Advancing the interoperability of electronic health records, as well as communication flows and interprofessional interaction within RAS, is essential to reduce the burden placed on patients to navigate services and carry clinical information. Furthermore, there is a clear need to strengthen the managerial function within BHU, with professionals that have health management training, protected time, ongoing professional development and technical support for planning, monitoring and network coordination. Lastly, problem-solving capacity in PHC must be expanded through multiprofessional teamwork, recognizing the increasing role of nursing and FCM, and the contribution of additional strategic professional groups - such as public health practitioners and other members of multidisciplinary teams.

## Data Availability

The sources of information used in the study are indicated in the body of the article.

## References

[1] Pearson-Stuttard J, Ezzati M, Gregg EW (2019). Multimorbidity-a defining challenge for health systems. Lancet Public Health.

[2] Gaal PA, Evetovits T, Sinko E, Davidovics K, Lam J (2025). Evaluation of the implementation experiences of the Hungarian integrated care coordination pilot. Health Policy.

[3] Rijken M, Struckmann V, van der Heide I.Hujala A.Barbabella F.van Ginneken E (2017). How to improve care for people with multimorbidity in Europe?.

[4] Macedo VLM, Sousa NP, Santos AC, Santos W, Stival MM, Rehem TCMSB (2025). Coordination of care in health systems for users with diabetes and hypertension a scoping review. Rev Latinoam Enferm.

[5] Almeida PF, Fausto MCR, Giovanella L (2011). Fortalecimento da atenção primária à saúde estratégia para potencializar a coordenação dos cuidados. Rev Panam Salud Pública.

[6] Rajan D, Rouleau K, Winkelmann J, Jakab M, Kringos D, Khalid F (2024). Implementing the primary health care approach: a primer..

[7] Duan-Porter W, Ullman K, Majeski B, Miake-Lye I, Diem S, Wilt TJ (2022). Care coordination models and tools-systematic review and key informant interviews. J Gen Intern Med.

[8] Giannotti EM, Louvison M, Chioro A (2025). Listas de espera na atenção ambulatorial especializada reflexões sobre um conceito crítico para o Sistema Único de Saúde. Cad Saúde Pública.

[9] Almeida PF, Medina MG, Fausto MCR, Giovanella L, Bousquat A, Mendonça MHM (2018). Coordenação do cuidado e atenção primária à saúde no Sistema Único de Saúde. Saúde Debate.

[10] Khatri R, Endalamaw A, Erku D, Wolka E, Nigatu F, Zewdie A (2023). Continuity and care coordination of primary health care a scoping review. BMC Health Serv Res.

[11] Melo EA, Gomes GG, Carvalho JO, Pereira PHB, Guabiraba KPL (2021). A regulação do acesso à atenção especializada e a Atenção Primária à Saúde nas políticas nacionais do SUS. Physis (Rio J.).

[12] Bousquat A, Giovanella L, Aquino R, Vilasbôas ALQ, Thumé E, Tomasi E (2026). Primary health care assessment in Brazil conception and methodology of the 2024 National Basic Health Unit Census. Cad Saúde Pública.

[13] Ministério da Saúde Questionário das UBS - instrumento para coleta dos dados..

[14] Fausto MCR, Giovanella L, Mendonça MHM, Seidl H, Gagno J (2014). A posição da Estratégia Saúde da Família na rede de atenção à saúde na perspectiva das equipes e usuários participantes do PMAQ-AB.. Saúde Debate.

[15] Souza MF, Santos AF, Reis IA, Santos MAC, Jorge AO, Machado ATGM (2017). Care coordination in PMAQ-AB an Item Response Theory-based analysis. Rev Saúde Pública.

[16] Instituto Brasileiro de Geografia e Estatística (2017). Classificação e caracterização dos espaços rurais e urbanos do Brasil: uma primeira aproximação.

[17] Asosega KA, Adebanji AO, Aidoo EN, Owusu-Dabo E (2024). Application of hierarchical/multilevel models and quality of reporting (2010-2020) a systematic review. ScientificWorldJournal.

[18] Bourget MMM, Cassenote AJF, Scheffer MC (2022). Physician turnover in primary health care services in the East Zone of São Paulo City, Brazil incidence and associated factors. BMC Health Serv Res.

[19] Shah SS, Safa A, Johal K, Obika D, Valentine S (2021). A prospective observational real world feasibility study assessing the role of app-based remote patient monitoring in reducing primary care clinician workload during the COVID pandemic. BMC Fam Pract.

[20] Bousquat A, Giovanella L, Facchini L, Mendonça MHM, Nedel FB, Cury G (2023). The Brazilian primary health care response to the COVID-19 pandemic individual and collective approaches. Front Public Health.

[21] Secretaria de Atenção Primária à Saúde, Ministério da Saúde Sumário Executivo Censo Nacional das Unidades Básicas de Saúde 2024..

[22] Kashiwakura HK, Gonçalves AO, Azevedo RR, Nunes A, Silva CAT (2021). Retrato da atenção básica no Brasil gastos e infraestrutura em municípios brasileiros. Ciênc Saúde Colet.

[23] Bousquat A, Giovanella L, Fausto MCR, Fusaro ER, Mendonça MHM, Gagno J (2017). Tipologia da estrutura das unidades básicas de saúde brasileiras os 5 R. Cad Saúde Pública.

[24] Bousquat A, Schenkman S, Almeida PF, Mota PHS, Giovanella L (2025). A estrutura das UBS brasileiras o que mudou entre 2012 e 2024?. Ciênc Saúde Colet.

[25] Ministério da Saúde (2013). Portaria Interministerial nº 1.369, de 8 de julho de 2013. Dispõe sobre a implementação do Projeto Mais Médicos para o Brasil.. Diário Oficial da União.

[26] Ministério da Saúde (2024). Portaria GM/MS nº 3.493, de 10 de abril de 2024. Altera a Portaria de Consolidação GM/MS nº 6, de 28 de setembro de 2017, para instituir nova metodologia de cofinanciamento federal do Piso de Atenção Primária à Saúde no âmbito do Sistema Único de Saúde (SUS).. Diário Oficial da União.

[27] Ministério da Saúde (2024). Portaria GM/MS nº 3.232, de 1º de março de 2024. Altera a Portaria de Consolidação GM/MS nº 5, de 28 de setembro de 2017, para instituir o Programa SUS Digital.. Diário Oficial da União.

[28] Celuppi IC, Mohr ETB, Felisberto M, Rodrigues TS, Hammes JF, Cunha CL (2024). Dez anos do Prontuário Eletrônico do Cidadão e-SUS APS em busca de um Sistema Único de Saúde eletrônico. Rev Saúde Pública.

[29] Ljungholm L, Edin-Liljegren A, Ekstedt M, Klinga C (2022). What is needed for continuity of care and how can we achieve it - Perceptions among multiprofessionals on the chronic care trajectory. BMC Health Serv Res.

[30] Mendes LS, Almeida PF (2020). Médicos da atenção primária e especializada conhecem e utilizam mecanismos de coordenação. Rev Saúde Pública.

[31] Lacerda RST, Almeida PF (2023). Coordenação do cuidado uma análise por meio da experiência de médicos da atenção primária à saúde. Interface (Botucatu).

[32] Miliauskas CR, Faus DP, Junkes L, Rodrigues RB, Junger W (2019). Associação entre internações psiquiátricas, cobertura de CAPS e atenção básica em regiões metropolitanas do RJ e SP, Brasil.. Ciênc Saúde Colet.

[33] Soto-Schulz K, Herrera-Echenique R, Brito-Díaz R, Pérez-Romero N (2025). Competencies required for the performance of primary health care managers a systematic review. Cad Saúde Pública.

[34] Oliveira LS (2025). Escopo de práticas da média gerência na atenção primária à saúde: uma análise a partir da experiência do município do Rio de Janeiro/RJ.

[35] Nunes FGS, Santos AM, Carneiro AO, Fausto MCR, Cabral LMS, Almeida PF (2022). Challenges to the provision of specialized care in remote rural municipalities in Brazil. BMC Health Serv Res.

[36] Almeida PF, Vilasbôas ALQ, Ribeiro AMVB, Silva AN, Casotti E (2024). Transition between primary and specialized care in monitoring systemic arterial hypertension restricted access and discontinuous care. Saúde Soc.

[37] Sousa MN, Oliveira BLCA, Alves MTSSB, Andrietta LS, Carvalho RHSBF (2024). Popular Health Clinics from the perspective of entrepreneurs and representatives of the medical profession. Saúde Soc.

[38] Oliveira CCRB, Silva EAL, Souza MKB (2021). Referral and counter-referral for the integrality of care in the Health Care Network. Physis (Rio J.).

[39] Vieira-Meyer APGF, Lavor ACH (2025). Do Ceará para o Brasil perspectiva histórica e desafios da experiência dos agentes comunitários de saúde - entrevista com Carlile Lavor. Ciênc Saúde Colet.

[40] Rede de Pesquisa em APS (2022). Bases para uma atenção primária à saúde integral, resolutiva, territorial e comunitária no SUS: aspectos críticos e proposições.

[41] McDonald KM, Schultz E, Albin L, Pineda N, Lonhart J, Sundaram V (2014). Care coordination atlas version 4..

[42] Allen LN, Rasanathan K, Mash R, Uribe MV, Martinez-Bianchi V, Kidd M (2025). Models of global primary care post-2030. Lancet Prim Care.

[43] Basu S, Berkowitz SA, Phillips RL, Bitton A, Landon BE, Phillips RS (2019). Association of primary care physician supply with population mortality in the United States, 2005-2015. JAMA Intern Med.

