# Supplementary material for: Care coordination through primary health care and network integration: the importance of ensuring comprehensive and integrated care in the Brazilian Unified National Health System
Source: Cad Saude Publica. 2026 Nov 2;42(Suppl 2):e00226625. doi: 10.1590/0102-311XEN226625 (PMC13730242; doi:10.1590/0102-311XEN226625)
Supplement: Translation

# Translation: Coordenação do cuidado pela atenção primária à saúde e integração à rede: a essencialidade de se garantir cuidados integrais e integrados no Sistema Único de Saúde

**DOI:** 10.1590/0102-311XPT226625

**Authors:** Patty Fidelis de Almeida, Simone Schenkman, Aylene Bousquat, Elisandréa Sguario Kemper, Ligia Giovanella

**Affiliations:** 1 Instituto de Saúde Coletiva, Universidade Federal Fluminense, Niterói, Brasil.; 2 Faculdade de Saúde Pública, Universidade de São Paulo, São Paulo, Brasil.; 3 Secretaria de Atenção Primária à Saúde, Ministério da Saúde, Brasília, Brasil.; 4 Centro de Estudos Estratégicos da Fiocruz Antonio Ivo de Carvalho, Fundação Oswaldo Cruz, Fiocruz, Rio de Janeiro, Brasil.

**Keywords:** Integração dos Serviços de Saúde, Prestação Integrada de Cuidados de Saúde, Atenção Primária à Saúde

## Abstract

A coordenação do cuidado e a integração da atenção primária à saúde (APS) à rede de atenção representam desafios estruturantes para a consolidação da integralidade no Sistema Único de Saúde (SUS). Este artigo analisa a prevalência do uso de instrumentos, estratégias e mecanismos de coordenação do cuidado e de regulação assistencial nas unidades básicas de saúde (UBS) e sua associação a fatores contextuais e estruturais. Foram analisados resultados do *Censo Nacional das Unidades Básicas de Saúde* (Censo das UBS) de 2024, referentes a 43.254 unidades com equipes de APS, a partir dos seguintes componentes: coordenação, integração à rede, mecanismos de regulação assistencial e cuidado compartilhado. A comunicação entre os serviços acontecia majoritariamente por meios informais, enquanto o uso de e-mail e prontuários eletrônicos interoperáveis era restrito. Os encaminhamentos via sistema de regulação ocorriam em cerca de dois terços das UBS, paralelamente a outros fluxos sob responsabilidade do usuário. Havia ampla disseminação do prontuário eletrônico nas UBS, porém o compartilhamento com outros pontos da rede era limitado. A articulação do cuidado mostrou-se maior entre UBS, Centros de Atenção Psicossocial e hospitais. A presença de gerentes nas UBS esteve associada à maior frequência, formalização da comunicação e adoção de instrumentos de coordenação. Os resultados sinalizam a necessidade de fortalecer políticas estruturantes que consolidem a APS como coordenadora do cuidado, com investimentos em infraestrutura digital, interoperabilidade dos sistemas de informação, qualificação da função gerencial e valorização do trabalho multiprofissional, de modo a ampliar a capacidade resolutiva dos serviços e reduzir as desigualdades socioespaciais.

## Introdução

Os sistemas de saúde enfrentam desafios crescentes, entre os mais relevantes estão as mudanças necessárias para o acompanhamento longitudinal de pessoas que vivem com enfermidades crônicas não transmissíveis e multimorbidade ^1^, sustentáveis a longo prazo ^2^. Adicionalmente, tais sistemas sofrem pressão contínua para a incorporação de tecnologias biomédicas que diversificam prestadores, o que pode contribuir para maior fragmentação do cuidado, exigindo maiores esforços de coordenação e integração entre serviços, profissionais, níveis assistenciais e outros setores - como a assistência social e os serviços comunitários ^2^.

Embora programas direcionados à gestão de doenças crônicas baseados em evidências tenham avançado, especialmente com a oferta de cuidados multiprofissionais, a ênfase em condições específicas limita a capacidade de respostas integrais e centradas nos usuários e nas famílias ^3^. Em sistemas de saúde orientados pela atenção primária à saúde (APS), espera-se que as equipes desempenhem a função de coordenar o cuidado ao longo do continuum assistencial ^4^. Em contextos nos quais a APS é coordenadora do cuidado, os usuários recebem atenção em saúde mais qualificada, expressa em planos terapêuticos singulares, no incentivo à participação ativa na tomada de decisões, na autogestão e em maior satisfação ^3^. Ainda assim, falhas na articulação entre os níveis assistenciais estão associadas a orientações em saúde contraditórias, à prescrição excessiva de medicamentos e ao aumento de hospitalizações ^3^.

Parte-se do pressuposto de que políticas e ações voltadas ao fortalecimento da APS a capacitam para coordenar o cuidado ^5^. Somente uma APS robusta é capaz de melhorar a eficiência e qualidade dos sistemas de saúde ao promover a continuidade do cuidado; ampliar a equidade e o acesso, especialmente para populações vulnerabilizadas; além de contribuir para a resiliência dos sistemas de saúde frente a crises sanitárias e emergências climáticas ^6^. De toda forma, *pari passu*, é premente o desenvolvimento e a implementação sistemática de estratégias e instrumentos que tornem possível o desempenho da coordenação ^7^, e que enfrente os problemas relacionados ao acesso e à organização da oferta de atenção especializada ^8^.

No Sistema Único de Saúde (SUS), a função de coordenação do cuidado é atribuída, sobretudo, às equipes da Estratégia Saúde da Família (ESF) ^5^. Neste artigo, a coordenação é compreendida como um processo intencional que busca estabelecer conexões entre serviços, ações e profissionais de saúde envolvidos no cuidado ofertado a usuários, familiares e territórios, com qualidade, valor elevado, em tempo oportuno, de forma contínua e integral ^9^. Argumenta-se que as possibilidades de coordenação pela APS são condicionadas, entre outros fatores, pela existência de políticas, práticas e mecanismos que viabilizam a integração entre diferentes níveis assistenciais, em estreita relação com processos de regulação assistencial ^9^. 

Mecanismos estruturados de comunicação e compartilhamento de informações que permitem reduzir repetições, melhorar a continuidade da atenção, apoiar decisões clínicas mais seguras e integrar diferentes serviços, especialmente para pessoas com condições crônicas complexas, são importantes instrumentos pró-coordenação ^10^. Já a regulação assistencial busca garantir que todos os recursos em saúde sejam distribuídos de forma justa, acessível e em tempo oportuno ^11^. Assim, sistemas de informação interoperáveis, protocolos, telessaúde e definição de fluxos nas Redes de Atenção à Saúde (RAS) -; aliados a estratégias de microrregulação da clínica pelas equipes de APS - são dispositivos imprescindíveis para operar processos de coordenação do cuidado e qualificar as demandas produzidas nos demais pontos do sistema de saúde ^11^.

Com base nos resultados do *Censo Nacional das Unidades Básicas de Saúde* (Censo das UBS) de 2024 ^12^, este artigo analisa a prevalência da utilização de instrumentos, estratégias e mecanismos de coordenação do cuidado e de regulação assistencial nas unidades básicas de saúde (UBS) brasileiras e sua associação a fatores contextuais (regiões, situação urbano-rural do município, porte populacional) e estruturais (presença de gerente, de médico com residência ou título de especialista em Medicina de Família e Comunidade - MFC - e número de equipes de saúde da família - eSF - na UBS). A partir de um amplo diagnóstico proveniente dos resultados do Censo das UBS, busca-se apresentar subsídios que orientem políticas públicas, organização de serviços e práticas em saúde direcionadas à coordenação do cuidado e integração da APS à rede.

## Metodologia

Foram analisados os resultados do Censo das UBS, realizado no ano de 2024. O instrumento de coleta de dados foi elaborado a partir de um conjunto de dimensões essenciais da APS, compreendida como integral, resolutiva, comunitária, territorial e articulada à rede, em consonância com o modelo assistencial da ESF, descritas em Bousquat et al. ^12^.

Neste artigo, foram examinados os resultados referentes às dimensões de coordenação do cuidado e da integração à rede assistencial e de cuidado compartilhado, presentes no instrumento ^13^. Para analisar a prevalência da utilização de instrumentos, estratégias e mecanismos de coordenação do cuidado e regulação assistencial nas UBS, foram selecionadas as seguintes variáveis: (1) instrumentos e frequência de comunicação entre as UBS e outros pontos da rede; (2) mecanismos de coordenação do cuidado e compartilhamento do prontuário eletrônico; (3) serviços com os quais as(os) profissionais da UBS compartilham cuidado; (4) modalidades de encaminhamento de usuários atendidos na UBS para consulta especializada; e (5) instrumentos e estratégias de regulação assistencial nas UBS.

Conforme o referencial adotado, para que a APS possa desempenhar a função de coordenadora do cuidado, é necessário o uso de instrumentos e mecanismos específicos para este fim, permitindo a comunicação interprofissional e o compartilhamento de informações clínicas, aliados a estratégias e processos que facilitem o acesso à retaguarda terapêutica ^9^ ^,^ ^14^. Ao mesmo tempo, embora na quase totalidade de municípios brasileiros as centrais de marcação de procedimentos especializados estejam presentes ^15^, os processos de microrregulação clínica são estratégicos para qualificar as referências realizadas pelas equipes de APS a partir de critérios de prioridade, monitoramento das filas e tempo de espera, a fim de garantir o acesso equitativo aos serviços especializados e hospitalares ^11^.

A coleta de dados ocorreu entre junho e setembro de 2024, por meio de procedimento eletrônico realizado no sistema e-Gestor Atenção Primária à Saúde (https://egestoraps.saude.gov.br/). Inicialmente, foram incluídos na base do Censo das UBS todos os 49.738 estabelecimentos cadastrados como de APS no Cadastro Nacional de Estabelecimentos de Saúde (CNES), em abril de 2024. Deste total, 4.026 estabelecimentos foram inativados pelos gestores municipais por não estarem em funcionamento no período de resposta ou por não se caracterizarem como serviços de APS, resultando em 45.712 estabelecimentos respondentes.

Após avaliação de consistência e completude do banco de dados, foram analisadas as informações de 44.938 estabelecimentos caracterizados como UBS. Esse total inclui também pontos de apoio às UBS que possuem CNES próprio, mas que não contavam com a presença regular de equipes.

Para fins da presente análise, foram excluídos estabelecimentos que não possuíam pelo menos uma eSF e/ou equipe de atenção primária (eAP) e/ou equipe de saúde da família ribeirinha (eSFR), considerando-se que, sem a presença de equipes, não há possibilidade de coordenação e integração à rede, totalizando 43.254 UBS com a seguinte distribuição pelas regiões do país: Norte = 3.722; Nordeste = 7.164; Sudeste = 12.956; Sul = 6.364; Centro-oeste = 3.048.

Para avaliar a associação com macrofatores, foram consideradas variáveis contextuais como: (a) região do país; (b) situação urbano-rural; e (c) porte populacional do município.

A caracterização da situação urbano-rural baseou-se na tipologia do Instituto Brasileiro de Geografia e Estatística (IBGE) ^16^, considerando-se como rurais e/ou remotos os municípios classificados como rural adjacente, rural remoto e intermediário remoto, correspondendo a 61,5% dos municípios e 15.451 UBS (35,7%). Os municípios urbanos e intermediários adjacentes corresponderam a 38,5% dos municípios e 27.803 UBS (64,3%).

O porte populacional foi definido conforme classificação do IBGE ^16^: pequeno porte I (até 20.000 habitantes); pequeno porte II (20.001 a 50.000 habitantes); médio porte (50.001 a 100.000 habitantes); grande porte (100.001 a 900.000 habitantes); e metrópoles (mais de 900.000 habitantes).

Como variáveis estruturais, foram consideradas: (a) presença de gerente na UBS, exercendo função exclusiva ou compartilhada com ações assistenciais (34.005 UBS; 78,6%); (b) presença de médico com residência ou título de especialista em MFC (12.855 UBS; 29,7%); (c) número de eSF na UBS (distribuídas da seguinte forma: 1 eSF: 30.042 [69,5%]; 2 eSF: 4.998 [11,6%]; 3 eSF: 1.974 [4,6%]; 4 ou mais eSF: 2.712 [6,3%]).

Para compreender o papel e o peso relativo de cada fator estrutural e contextual nos resultados, foram realizadas análises bivariadas e multivariadas por meio de regressão logística multinível. Considerou-se o contexto municipal para as variáveis relativas às modalidades de encaminhamento para consulta especializada, marcadas e informadas na hora, ou posteriormente, ao usuário pela UBS; e as referentes aos instrumentos e estratégias de regulação assistencial (protocolos técnicos, listas de usuários, encaminhamentos por enfermeiras(os), profissionais responsáveis pela regulação e monitoramento do tempo de espera dos usuários). Desta forma, todas as variáveis foram ajustadas mutuamente.

Para estas análises, são apresentados os *odds ratios* (OR), os intervalos de 95% de confiança (IC95%) e valores de p, além da constante para o nível municipal e o coeficiente de correlação intraclasse, denotando a importância de considerar a análise multinível ^17^ no efeito das variáveis locorregionais sobre as modalidades de encaminhamento do usuário e as estratégias de regulação assistencial.

As análises foram realizadas com o programa Stata, versão 14.2 (https://www.stata.com).

O estudo do Censo das UBS foi aprovado pelo Comitê de Ética em Pesquisa da Faculdade de Saúde Pública, Universidade de São Paulo (FSP/USP; CAAE 78767024.2.0000.5421 e parecer 6.779.222).

## Resultados

Os principais instrumentos para a comunicação entre os profissionais da UBS e outros pontos da rede foram mensagens por WhatsApp (66,7%) - aplicativo de mensagens de texto e áudio especificado no instrumento do Censo das UBS -, e o contato telefônico (62,8%), sendo a comunicação via e-mail menos frequente (33,5%). O uso combinado dos três instrumentos foi relatado por 26,8% das UBS, enquanto 21,2% não utilizavam nenhum dos meios. Ainda que a maioria das UBS utilizasse algum instrumento, a comunicação era frequente em menos da metade das UBS (41%). As regiões Norte e Nordeste apresentaram os piores resultados, enquanto Sul e Sudeste registraram maior diversificação e frequência. A Região Centro-oeste apresentou resultados intermediários, mais próximos ao padrão nacional (Tabela 1). 

**Tabela 1:** Instrumentos e frequência da comunicação entre os profissionais das unidades básicas de saúde (UBS) e de outros pontos da rede, por grandes regiões *, Brasil, municípios rurais e urbanos e presença de gerente **, 2024.

Instrumentos/Frequência	Norte	Nordeste	Sudeste	Sul	Centro-oeste	Brasil	Rural e/ou remoto	Urbano e intermediários adjacentes	Sem gerência	Com gerência
	n (%)	n (%)	n (%)	n (%)	n (%)	n (%)	n (%)	n (%)	n (%)	n (%)
WhatsApp	2.151 (57,8)	10.386 (60,5)	9.233 (71,3)	4.968 (78,1)	2.123 (69,7)	28.861 (66,7)	9.985 (64,6)	18.876 (67,9)	6.226 (67,5)	22.625 (66,5)
Contato telefônico	1.747 (46,9)	8.449 (49,2)	9.935 (76,7)	5.049 (79,3)	1.963 (64,4)	27.143 (62,8)	8.381 (54,2)	18.762 (67,5)	5.507 (59,7)	21.629 (63,6)
E-mail	547 (14,7)	2.686 (15,7)	6.883 (53,1)	3.505 (55,1)	846 (27,8)	14.467 (33,5)	3.039 (19,7)	11.428 (41,1)	2.496 (27,0)	11.965 (35,2)
Utiliza as três formas	439 (11,8)	1.993 (11,6)	5.545 (42,8)	2.935 (46,1)	667 (21,9)	11.579 (26,8)	2.430 (15,7)	9.149 (32,9)	2.043 (22,1)	9.532 (28,0)
Nenhuma das três formas	1.233 (33,1)	5.060 (29,5)	1.678 (12,9)	620 (9,7)	563 (18,5)	9.154 (21,2)	3.954 (25,6)	5.200 (18,7)	2.007 (21,8)	7.140 (21,0)
Frequência da comunicação (sempre/quase sempre)	1.601 (43,0)	6.444 (37,6)	5.988 (46,2)	2.721 (42,8)	1.254 (41,1)	18.008 (41,6)	6.317 (40,9)	11.691 (42,1)	3.022 (32,7)	14.979 (44,1)
Totais de UBS por região/contexto	3.722 (8,6)	17.164 (39,7)	12.956 (29,9)	6.364 (14,7)	3.048 (7,1)	43.254 (100,0)	15.451 (35,7)	27.803 (64,3)	9.231 (21,3)	34.005 (78,6)

Fonte: Censo das UBS ^12^.

* Número de respostas: Brasil = 43.254; Norte = 3.722; Nordeste = 17.164; Sudeste = 12.956; Sul = 6.364; Centro-oeste = 3.048; (*missing* = 2);

** Gerente (*missing* = 18).

As desigualdades no uso dos instrumentos de comunicação foram observadas também entre municípios urbanos e rurais. Os urbanos registraram maior uso de telefone (67,5%) e e-mail (41,1%); entre os municípios rurais, apenas 15,7% utilizavam os três instrumentos de comunicação. A presença de gerência na UBS esteve associada a maior utilização do telefone, e-mail e combinação dos três instrumentos, bem como a maior frequência de comunicação (“sempre/quase sempre”). Cabe destacar que de 10% a 30% das UBS, dependendo da região, não utilizavam nenhum dos três instrumentos apresentados, com menores frequências nas UBS de regiões Norte/Nordeste, de contexto rural e com ausência de gerência (Tabela 1).

Os resultados do Censo das UBS revelaram elevada utilização do prontuário eletrônico na APS brasileira (88,7% das UBS). O compartilhamento dos registros com algum outro ponto de atenção da rede, para fins de comunicação e coordenação do cuidado, ocorria em 50,8% das UBS, com grandes diferenças regionais (Sul: 73,5%; Norte: 31,3%). Alerta-se que o compartilhamento do prontuário ocorria principalmente com outras UBS (70,9%). Contudo, para a continuidade assistencial com outros serviços da RAS, a realização de tal compartilhamento não alcançava um quarto dos estabelecimentos, com percentuais variados a depender dos serviços: Centros de Atenção Psicossocial (CAPS; 21,1%), serviços especializados públicos (18,8%), serviços de urgência/Unidade de Pronto Atendimento (UPA; 18,4%) e Centro de Especialidades Odontológicas (CEO; 17,7%). Percentuais ainda mais baixos foram encontrados em relação aos laboratórios e hospitais públicos (Tabela 2). 

**Tabela 2:** Estratégias de coordenação do cuidado e compartilhamento do prontuário eletrônico entre níveis assistenciais nas unidades básicas de saúde (UBS), por grandes regiões *, Brasil, municípios rurais e urbanos e presença de gerente **, 2024.

	Norte	Nordeste	Sudeste	Sul	Centro-oeste	Brasil	Rural e/ou remoto	Urbano e intermediários adjacentes	Sem gerência	Com gerência
	n (%)	n (%)	n (%)	n (%)	n (%)	n (%)	n (%)	n (%)	n (%)	n (%)
Estratégias de coordenação										
Reuniões técnicas ou sessões clínicas conjuntas	1.766 (47,5)	8.037 (46,8)	7.994 (61,7)	3.562 (56,0)	1.317 (43,2)	22.676 (52,4)	7.178 (46,5)	15.498 (55,7)	4.261 (46,2)	18.407 (54,1)
Consulta compartilhada	787 (21,1)	5.107 (29,8)	4.029 (31,1)	1.782 (28,0)	768 (25,2)	12.473 (28,8)	3,922 (25,4)	8.551 (30,8)	2.235 (24,2)	10.234 (30,1)
Plano de cuidado compartilhado/PTS	400 (10,8)	3.255 (19,0)	1.097 (8,5)	1.919 (30,2)	396 (13,0)	7.041 (16,3)	1.874 (12,1)	5.624 (20,2)	1.303 (14,1)	6.194 (18,2)
Telessaúde	407 (9,9)	3.317 (18,7)	1.120 (8,4)	1.947 (29,5)	370 (12,1)	7.163 (15,9)	2.493 (16,1)	4.548 (16,4)	1.202 (13,0)	5.839 (17,2)
Ficha de referência contrarreferência	1.876 (50,4)	8.512 (49,6)	8.354 (64,5)	3.611 (56,7)	1.596 (52,4)	23.949 (55,4)	7.106 (46,0)	16.843 (60,6)	4.697 (50,9)	19.246 (56,6)
Recebe resumo de alta hospitalar (sempre/quase sempre)	760 (20,4)	3.714 (21,6)	5.454 (42,1)	1.732 (27,2)	584 (19,2)	12.244 (28,3)	3.698 (23,9)	8.546 (30,7)	2.028 (22,0)	10.211 (30,0)
Comunicação por PEC com profissionais de outros pontos da rede	1.164 (31,3)	6.842 (39,9)	7.521 (58,1)	4.680 (73,5)	1.773 (58,2)	21.980 (50,8)	6.294 (40,7)	15.686 (56,4)	4.197 (45,5)	17.782 (52,3)
Serviços com os quais as UBS compartilham o PEC										
Outras UBS	1.694 (45,5)	11.105 (64,7)	9.876 (76,2)	5.457 (85,8)	2.541 (83,4)	30.673 (70,9)	9.506 (61,5)	21.167 (76,1)	6.384 (69,2)	24.280 (71,4)
CAPS	282 (7,6)	1.257 (7,3)	3.716 (28,7)	3.246 (51,0)	635 (20,8)	9.136 (21,1)	914 (5,9)	8.222 (29,6)	1.541 (16,7)	7.592 (22.3)
Serviços especializados públicos	205 (5,5)	1.357 (7,9)	3.706 (28,6)	2.270 (35,7)	599 (19,7)	8.137 (18,8)	1.019 (6,6)	7.118 (25,6)	1.356 (14,7)	6.777 (19,9)
Prontos-socorros e outros serviços de urgência/UPA	188 (5,0)	834 (4,9)	3.367 (26,0)	2.765 (43,5)	810 (26,6)	7.964 (18,4)	1.170 (7,6)	6.794 (24,4)	1.316 (14,3)	6.645 (19,5)
CEO	316 (8,5)	1.768 (10,3)	3.084 (23,8)	1.976 (31,0)	502 (16,5)	7.646 (17,7)	592 (3,8)	7.054 (25,4)	1.207 (13,1)	6.437 (18,9)
Serviços exames/laboratoriais	215 (5,8)	896 (5,2)	2.772 (21,4)	1.718 (27,0)	696 (22,8)	6.297 (14,6)	939 (6,1)	5,358 (19,3)	989 (10,7)	5.307 (15,6)
Hospitais da rede pública	125 (3,4)	615 (3,6)	1.234 (9,5)	597 (9,4)	423 (13,9)	2.994 (6,9)	606 (3,9)	2,388 (8,6)	449 (4,9)	2.543 (7,5)
Totais de UBS por região/contexto	3.722 (8,6)	17.164 (39,7)	12.956 (29,9)	6.364 (14,7)	3.048 (7,1)	43.254 (100,0)	15.451 (35,7)	27.803 (64,3)	9.231 (21,3)	34.005 (78,6)

CAPS: Centro de Atenção Psicossocial; CEO: Centro de Especialidades Odontológicas; PEC: Prontuário Eletrônico Compartilhado; PTS: Projeto Terapêutico Singular; UPA: unidade de pronto-atendimento.

Fonte: Censo das UBS ^12^.

* Número de respostas: Brasil = 43.254; Norte = 3.722; Nordeste = 17.164; Sudeste = 12.956; Sul = 6.364; Centro-oeste = 3.048; (*missing* = 2);

** Gerente (*missing* = 18).

Dentre as estratégias e mecanismos de coordenação examinados na Tabela 2, as reuniões técnicas/sessões clínicas conjuntas (52,4%) e as fichas de referência/contrarreferência (55,4%) foram as mais consolidadas. Entretanto, o Projeto Terapêutico Singular (PTS), a telessaúde (teleconsultoria), a consulta compartilhada e o recebimento regular de resumo de alta hospitalar apresentaram baixa frequência. Os melhores desempenhos foram observados no Sudeste em relação à realização de sessões clínicas conjuntas (61,7%) e recebimento de resumo de alta hospitalar (42,1%) e no Sul quanto ao uso do PTS e telessaúde - em torno de 30%. A ausência de algum mecanismo de coordenação alcançou quase 10% das UBS, com piores resultados no Norte. Municípios urbanos e UBS com gerência apresentaram maior adoção de mecanismos de coordenação e integração via prontuário eletrônico com serviços dos demais pontos da RAS (Tabela 2).

Os resultados do Censo das UBS mostraram que os serviços com os quais os profissionais da UBS compartilham o cuidado com maior frequência são os CAPS (74,6%) e hospitais (67%). Em cerca de 50% das UBS, tais serviços se relacionavam com maternidades, CEO e serviços ambulatoriais de especialidades e de infecções sexualmente transmissíveis (IST). Coordenação menos frequente foi observada em relação aos serviços de reabilitação e policlínicas. Observou-se forte desigualdade regional, com a Região Sudeste apresentando maior integração para quase todos os serviços listados, enquanto as regiões Norte e Nordeste apresentaram as menores, exceto para as policlínicas. As disparidades rurais-urbanas foram bastante expressivas, especialmente para serviços de saúde mental, CEO, atenção ambulatorial, IST e reabilitação. A presença de gerência associou-se sistematicamente ao maior nível de compartilhamento de cuidado relacionado a todos os serviços especializados (Tabela 3).

**Tabela 3:** Serviços com os quais as(os) profissionais das unidades básicas de saúde (UBS) compartilham o cuidado, por grandes regiões *, Brasil, municípios rurais e urbanos e presença de gerente **, 2024.

Tipo de serviço	Norte	Nordeste	Sudeste	Sul	Centro-oeste	Brasil	Rural e/ou remoto	Urbano e intermediários adjacentes	Sem gerência	Com gerência
	n (%)	n (%)	n (%)	n (%)	n (%)	n (%)	n (%)	n (%)	n (%)	n (%)
CAPS	2.451 (65,8)	12.473 (72,7)	10.721 (82,7)	4.635 (72,8)	1.975 (64,8)	32.255 (74,6)	8.588 (55,6)	23.667 (85,1)	6.597 (71,5)	25.645 (75,4)
Hospitais	2.453 (65,9)	11.950 (69,6)	8.468 (65,4)	4.145 (65,1)	1.986 (65,2)	29.002 (67,0)	10.586 (68,5)	18.416 (66,2)	5.863 (63,5)	23.129 (68,0)
Maternidades	1.701 (45,7)	9.032 (52,6)	7.536 (58,2)	3.441 (54,1)	1.121 (36,8)	22.831 (52,8)	6.634 (42,9)	16.197 (58,3)	4.231 (45,8)	18.595 (54,7)
CEO	1.296 (34,8)	8.432 (49,1)	7.547 (58,3)	3.211 (50,5)	1.343 (44,1)	21.829 (50,5)	4.964 (32,1)	16.865 (60,7)	4.089 (44,3)	17.737 (52,2)
Centro ambulatorial de especialidades	1.122 (30,1)	7.145 (41,6)	8.085 (62,4)	3.511 (55,2)	1.337 (43,9)	21.200 (49,0)	5.383 (34,8)	15.817 (56,9)	4.049 (43,9)	17.145 (50,4)
Serviços de atenção especializada em IST	2.083 (56,0)	6.869 (40,0)	7.886 (60,9)	3.744 (58,8)	1.510 (49,6)	22.092 (51,1)	4.796 (31,0)	17.296 (62,2)	3.981 (43,1)	18.102 (53,2)
Policlínicas regionais	925 (24,9)	7.483 (43,6)	3.897 (30,1)	1.355 (21,3)	699 (22,9)	14.359 (33,2)	4.998 (32,3)	9.361 (33,7)	2.853 (30,9)	11.503 (33,8)
Centros especializados de reabilitação	706 (19,0)	4.067 (23,7)	4.532 (35,0)	1.609 (25,3)	789 (25,9)	11.703 (27,1)	2.906 (18,8)	8.797 (31,6)	1.934 (20,9)	9.769 (28,7)
Totais de UBS por região/contexto	3.722 (8,6)	17.164 (39,7)	12.956 (29,9)	6.364 (14,7)	3.048 (7,1)	43.254 (100,0)	15.451 (35,7)	27.803 (64,3)	9.231 (21,3)	34.005 (78,6)

CAPS: Centro de Atenção Psicossocial; CEO: Centro de Especialidades Odontológicas; IST: infecções sexualmente transmissíveis.

Fonte: Censo das UBS ^12^.

* Número de respostas: Brasil = 43.254; Norte = 3.722; Nordeste = 17.164; Sudeste = 12.956; Sul = 6.364; Centro-oeste = 3.048; (*missing* = 2);

** Gerente (*missing* = 18).

Entre as UBS brasileiras, coexistem múltiplas formas para o encaminhamento dos usuários para consultas especializadas. A mais consolidada foi a marcação via sistema de regulação, com data informada posteriormente (59,6%) ou no momento (27,9%), predominante no Sudeste e Sul e em áreas urbanas. Modalidades que responsabilizam o usuário pela busca da consulta especializada, como o recebimento da ficha de referência e indicação de busca por um serviço informado pela equipe, foram expressivas, chegando a aproximadamente 50% nas regiões Norte, Nordeste, Centro-Oeste e em municípios rurais. Na mesma direção, 7% das UBS indicavam ao usuário buscar a consulta especializada por conta própria. A presença de médicos com especialização ou residência em MFC, de gerência e de maior número de eSF aumentaram a proporção de consultas marcadas pela UBS (Tabela 4). 

**Tabela 4:** Modalidades de encaminhamento de usuários atendidos nas unidades básicas de saúde (UBS) para consulta especializada, por grandes regiões *, Brasil, municípios rurais e urbanos, presença de médico com residência ou título de especialista em Medicina de Família e Comunidade (MFC) e de gerente na UBS, 2024.

Modalidades de encaminhamento	Consulta marcada pela UBS via sistema de regulação e data posteriormente informada		Usuário recebe ficha de referência e se dirige ao serviço indicado pela equipe		Consulta marcada pelo usuário junto à central de marcação/regulação		Consulta marcada pela UBS e informada na hora ao usuário		Usuário busca consulta especializada por conta própria		Consulta marcada pela UBS e informada na hora ou depois ao usuário **	
	n	%	n	%	n	%	n	%	n	%	n	%
Macrorregiões												
Norte	2.037	54,7	1.918	51,5	1.150	30,9	802	21,6	262	7,0	2.342	62,9
Nordeste	7.999	46,6	8.321	48,5	8.542	49,8	4.470	26,0	1.522	8,9	9.915	57,8
Sudeste	9.565	73,8	4.909	37,9	3.077	23,8	4.287	33,1	735	5,7	10.629	82,0
Sul	4.531	71,2	2.449	38,5	1.684	26,5	1.872	29,4	305	4,8	4.943	77,7
Centro-oeste	1.628	53,4	1.589	52,1	1.057	34,7	656	21,5	156	5,1	1.850	60,7
Brasil	25.760	59,6	19.186	44,4	15.510	35,9	12.087	27,9	2.980	6,9	29.679	68,6
Situação rural-urbana												
Rural e/ou remoto	6.476	41,8	8.176	52,9	7.037	45,5	3.697	23,9	1,378	8,9	8.087	52,3
Urbano e intermediários adjacentes	19.284	69,4	11.010	39,6	8.473	30,5	8.390	30,2	1.602	5,8	21.592	77,7
Médico com residência/especialização em MFC ***												
Não	17.133	56,4	13.946	45,9	11.501	37,9	8.196	27,0	2.191	7,2	20.024	65,9
Sim	8.620	67,1	5.235	40,7	4.006	31,2	3.890	30,3	788	6,1	9.647	75,0
Presença de gerente												
Não	4.767	51,6	4.111	44,5	3.942	42,7	2.295	24,9	701	7,6	5.700	61,8
Sim	20.982	61,7	15.069	44,3	11.565	34,0	9.787	28,8	2.279	6,7	23.967	70,5
Porte populacional												
Grande porte/Metrópole	10.032	85,7	3.712	31,7	2.246	19,2	3.760	32,1	553	4,7	10.585	90,4
Médio porte/Pequeno porte	15.728	49,9	15.474	49,1	13.264	42,0	8.327	26,4	2.427	7,7	19,094	60,5
Número de eSF ^#^												
1	16.308	54,3	14.009	46,6	11.971	39,9	7.945	26,5	2.237	7,4	19.245	64,1
2	3.463	69,3	2.090	41,8	1.485	29,7	1.396	27,9	280	5,6	3.834	76,7
3	1.571	79,6	686	34,8	437	22,1	639	32,4	101	5,1	1.682	85,2
4 ou mais	2.105	77,6	1.009	37,2	617	22,7	1.009	37,2	135	5,0	2.295	84,6
Consulta marcada pela UBS e informada na hora ou depois ao usuário	Análise bivariada						Análise multivariada					
	OR			IC95%			OR			IC95%		
Grande porte populacional/Metrópole	6,12			5,73-6,53			8,22			6,15-11,00		
Macrorregião Sudeste	2,70			2,56-2,84			2,86			2,44-3,36		
Contexto urbano e intermediários adjacentes	3,17			3,03-3,30			2,03			1,74-2,36		
Presença de gerente na UBS	1,48			1,41-1,55			1,31			1,19-1,43		
Médico com residência/especialização em MFC	1,56			1,48-1,63			1,11			1,03-1,20		
Número de eSF	1,36			1,33-1,39			1,05			1,01-1,10		
Constante	-			-			0,88			0,77-1,00		
Contexto municipal	-			-			5,22			4,83-5,64		
ICC	-			-			0,61			0,59-0,63		
LR												
n	43.186-43.252						43.163					
p	< 0,0001						< 0,0001					

eSF: equipe de saúde da família; ICC: coeficiente de correlação intraclasse; IC95%: intervalo de 95% de confiança; LR: *likelihood ratio*; OR: *odds ratio*.

Fonte: Censo das UBS ^12^.

* Número de respostas com equipes: Brasil = 43.254; Norte = 3.722; Nordeste = 17.164; Sudeste = 12.956; Sul = 6.364; Centro-oeste = 3.048; (*missing* = 2);

** Consultas marcadas exclusivamente nestas modalidades 39,7% das UBS brasileiras (17.179);

*** Médicos com título de MFC (*missing* = 17);

^#^ UBS de acordo com o número de eSF (*missing* = 66).

Observou-se gradiente importante para o agendamento de consultas pela UBS via sistema de regulação, com maiores chances de ocorrência, mesmo após os ajustes no modelo multivariado, em municípios de grande porte populacional ou metrópoles (8,22 vezes); na Região Sudeste (2,86 vezes); em contexto urbano (2,03 vezes), com a presença de gerência na unidade (1,31 vez), com médico especialista em MFC (1,11 vez) e com maior número de eSF (1,08 vez). O contexto municipal mostrou-se relevante para a forma de marcação de consultas que beneficiam o usuário, com OR de 5,22 e coeficiente de correlação intraclasse (ICC, acrônimo em inglês) de 61% (Tabela 4)

Os instrumentos de regulação assistencial mais prevalentes nas UBS foram protocolos técnicos para acesso à atenção especializada (67,8%) e registro/lista de usuários encaminhados (57,7%), com melhor desempenho na Região Sudeste e nos municípios com mais de 100 mil habitantes (grande porte/metropolitanos). Encaminhamentos solicitados por enfermeiros estiveram presentes em aproximadamente metade das UBS. Estratégias como o monitoramento do tempo de espera (34,9%) e designação de profissional responsável pela regulação na UBS (35,2%) foram menos frequentes. Destacou-se que todos os dispositivos foram mais frequentes em municípios urbanos, em UBS com gerência e com maior número de eSF, seguido por serviços com médico com residência ou especialista em MFC

Observou-se gradiente importante para os instrumentos e estratégias de regulação assistencial, persistindo após os ajustes do modelo multivariado, com maiores chances de ocorrência em municípios com grande porte populacional ou metrópoles, especialmente para encaminhamentos realizados por enfermeiras e pelo uso de protocolos (2,34-3,10 vezes) e de acordo com as macrorregiões: Região Sudeste, para o monitoramento do tempo de espera (1,91 vez) e o registro/lista de usuários (1,43 vez); Região Sul, para os protocolos técnicos (1,97 vez); Região Nordeste, para os encaminhamentos realizados por enfermeiras (2,06 vezes); e Região Norte, para profissional responsável pela regulação (1,28 vez). 

Também se observou associação com o contexto urbano para os encaminhamentos solicitados por enfermeiros, para a lista de usuários e especialmente para o uso de protocolos (1,15-1,88 vez). Porém, o contexto urbano ajustado deixou de ser significativo para a existência de profissionais responsáveis pela regulação assistencial e para o monitoramento do tempo de espera dos usuários. Ainda, o contexto municipal mostrou-se relevante, especialmente para a estratégia de encaminhamentos solicitados por enfermeiros, com OR de 3,54 e ICC de 52% mais prevalente na Região Nordeste. As demais estratégias apresentaram OR de 2,00-2,46, com ICC variando de 38-43%.

A presença de gerência na unidade foi significativa, principalmente para o monitoramento do tempo de espera dos usuários e para a presença de profissional responsável pela regulação (1,81-2,14 vezes), com a existência de pelo menos um médico especialista em MFC na UBS (1,15-1,26 vez) e com maior número de eSF (1,04-1,10 vez) - ambos com maior associação ao uso de protocolos técnicos e à existência de profissional responsável pela regulação (Tabela 5).

**Tabela 5:** Instrumentos e estratégias de regulação assistencial nas unidades básicas de saúde (UBS), por grandes regiões *, Brasil, municípios rurais e urbanos, presença de médico com residência ou título de especialista em Medicina de Família e Comunidade (MFC) e de gerente, 2024.

Estratégias de regulação	Protocolos técnicos para acesso aos serviços de atenção especializada		Registro/Lista dos usuários encaminhados para outros pontos de atenção da rede		Encaminhamentos solicitados por enfermeiras/os		Profissional responsável pela regulação assistencial nas UBS		Monitoramento dos tempos de espera dos usuários encaminhados a outros pontos de atenção da rede	
	n	%	n	%	n	%	n	%	n	%
Macrorregiões										
Norte	2.423	65,1	2.030	54,5	1.822	48,9	1.566	42,1	1.215	32,6
Nordeste	9.853	57,4	8.899	51,9	9.697	56,5	5.223	30,4	5.205	30,3
Sudeste	10.046	77,5	8.877	68,5	6.417	49,5	5.346	41,3	5,582	43,1
Sul	4.934	77,5	3.748	58,9	3.432	53,9	2.140	33,6	2.327	36,6
Centro-oeste	2.090	68,6	1.410	46,3	1.279	42,0	968	31,8	785	25,8
Brasil	29.346	67,8	24.964	57,7	22.646	52,4	15.243	35,2	15.114	34,9
Presença de gerente										
Não	5.123	55,5	4.477	48,5	4.152	45,0	2.172	23,5	2.280	24,7
Sim	24.211	71,2	20.476	60,2	17.484	54,4	13.063	38,4	12.826	37,7
Médicos com residência ou especialização em MFC **										
Não	19.760	65,0	16.987	55,9	15.306	50,4	10.087	33,2	9.939	32,7
Sim	9.576	74,5	7.969	62,0	7.335	57,1	5.153	40,1	5.169	40,2
Situação rural-urbana										
Rural e/ou remoto	8.525	55,2	7.505	48,6	7.059	45,7	4.745	30,7	4.368	28,3
Urbano e intermediários adjacentes	20.821	74,9	17.459	62,8	15.587	56,1	10.498	37,8	10.746	38,7
Porte populacional										
Grande porte/Metrópole	9.863	84,2	7.968	68,0	7.553	64,5	5.140	43,9	5.338	46,0
Médio porte/Pequeno porte	19.483	61,8	16.996	53,9	15.093	47,9	10.103	32,0	9.726	30,8
Número de eSF ***										
1	19.100	63,6	16.631	55,4	15.193	50,6	9.770	32,5	9.679	32,2
2	3.744	74,9	3.024	60,5	2.714	54,3	1.974	39,5	1.874	37.5
3	1.628	82,5	1.301	65,9	1.182	59,9	820	41,5	878	44,5
4 ou mais	2.320	85,6	1.908	70.4	1.792	66,1	1.404	51,8	1.384	51,0
Análise bivariada	OR	IC95%	OR	IC95%	OR	IC95%	OR	IC95%	OR	IC95%
Grande porte populacional/Metrópole	3,30	3,12-3,49	1,82	1,74-1,90	1,98	1,89-2,07	1,66	1,59-1,73	1,91	1,83-2,00
Macrorregião	1,76	1,66-1,88	1,92	1,84-2,01	1,32	1,27-1,37	1,37	1,28-1,47	1,65	1,58-1,72
Presença de gerente	1,98	1,89-2,08	1,61	1,53-1,68	1,46	1,39-1,53	2,03	1,92-2,14	1,85	1,75-1,95
Contexto urbano e intermediários adjacentes	2,42	2,32-2,53	1,79	1,72-1,86	1,52	1,46-1,58	1,37	1,31-1,43	1,60	1,53-1,67
Médico com título especialista MFC	1,57	1,50-1,64	1,29	1,23-1,34	1,31	1,26-1,36	1,35	1,29-1,40	1,38	1,33-1,44
Número de eSF	1,36	1,33-1,40	1,18	1,16-1,21	1,20	1,17-1,22	1,23	1,21-1,26	1,23	1,20-1,25
LR										
n	43.185-43.249		43.184-43.248		43.185-43.249		43.185-43.249		43.183-43.247	
p	< 0,0001		< 0,0001		< 0,0001		< 0,0001		< 0,0001	
Análise multivariada	OR	IC95%	OR	IC95%	OR	IC95%	OR	IC95%	OR	IC95%
Grande porte populacional/Metrópole	3,10	2,02-2,29	1,49	1,24-1,80	2,34	1,85-2,97	1,51	1,23-1,85	1,73	1,43-2,09
Macrorregião	1,97	1,71-2,26	1,91	1,71-2,13	2,06	1,81-2,34	1,28	1,06-1,56	1,43	1,27-1,60
Presença de gerente	1,79	1,66-1,92	1,63	1,52-1,74	1,58	1,46-1,70	2,14	1,97-2,31	1,81	1,67-1,95
Contexto urbano e intermediários adjacentes	1,88	1,68-2,11	1,40	1,26-1,56	1,15	1,01-1,31	0,94	0,84-1,05	1,11	0,99-1,24
Médico com título especialista MFC	1,24	1,17-1,32	1,17	1,10-1,23	1,15	1,09-1,22	1,26	1,19-1,33	1,19	1,12-1,26
Número de eSF	1,10	1,06-1,15	1,05	1,01-1,08	1,04	1,01-1,07	1,09	1,06-1,13	1,04	1,01-1,07
Constante	0,72	0,65-0,79	0,55	0,50-0,61	0,36	0,32-0,40	0,23	0,22-0,25	0,16	0,15-0,18
Contexto municipal	2,42	2,23-2,62	2,00	1,85-2,17	3,54	3,29-3,82	2,46	2,27-2,66	2,10	1,93-2,29
ICC	0,42	0,40-0,44	0,38	0,36-0,40	0,52	0,50-0,54	0,43	0,41-0,45	0,39	0,37-0,41
LR										
n	43.163		43.162		43.163		43.163		43.161	
p	< 0,0001		< 0,0001		< 0,0001		< 0,0001		< 0,0001	

eSF: equipe de saúde da família; ICC: coeficiente de correlação intraclasse; IC95%: intervalo de 95% de confiança; LR: *likelihood ratio*; OR: *odds ratio*.

Fonte: Censo das UBS ^12^.

* Número de respostas com equipes: Brasil = 43.254; Norte = 3.722; Nordeste = 17.164; Sudeste = 12.956; Sul = 6.364; Centro-oeste = 3.048; (*missing* = 5-7);

** Médicos com título de MFC (*missing* = 17);

*** UBS de acordo com o número de eSF (*missing* = 66).

## Discussão 

A coordenação do cuidado a partir da APS e sua integração às redes de atenção à saúde constituem um dos principais desafios dos sistemas públicos universais ^6^. Superar essa fragmentação é essencial para garantir atenção integral e integrada, capaz de responder adequadamente às mudanças demográficas e epidemiológicas ^3^. A organização da rede assistencial a partir da APS é uma condição fundamental para assegurar o acesso equitativo conforme as necessidades da população e efetivar o princípio da integralidade à saúde ^9^.

Nas UBS brasileiras, o aplicativo WhatsApp e o telefone são os instrumentos mais citados para comunicação entre profissionais da RAS, enquanto o e-mail, que permite registros mais formais e rastreáveis, permaneceu residual nas regiões Norte, Nordeste e Centro-oeste, sendo o uso combinado dos três instrumentos baixo. A prevalência de meios informais, associada à rotatividade profissional ^18^, pode comprometer a institucionalidade e a continuidade da comunicação interprofissional. Aplicativos móveis e o telefone passaram a ser amplamente utilizados para a comunicação e o monitoramento em saúde, sobretudo a partir da pandemia de COVID-19 ^19^, contudo, no país, a maioria dos profissionais utilizou o telefone pessoal para comunicação com usuários e outros profissionais ^20^. Segundo dados do Censo das UBS, apenas 24,8% das UBS dispõem de celular institucional e, embora o acesso à internet tenha melhorado, sua qualidade em parte das UBS ainda é insuficiente, com piores resultados nas regiões Norte e Nordeste ^21^. Assim, torna-se urgente investir na disponibilização de dispositivos móveis institucionais com planos de dados como parte do instrumental de trabalho dos profissionais, a fim de favorecer a comunicação interprofissional e o acompanhamento contínuo dos usuários. 

As desigualdades territoriais na disponibilidade de instrumentos para comunicação são expressivas. Sistematicamente, as regiões Sul e Sudeste alcançaram melhores resultados, convergentes a estudos que apontam que regiões com piores indicadores socioeconômicos, como produto interno bruto (PIB) *per capita* e o Índice de Desenvolvimento Humano Municipal (IDH-M), especialmente o Norte e o Nordeste, concentram tipologias de infraestrutura das UBS classificadas como reprovadas, rudimentares e restritas ^22^ ^,^ ^23^. De toda forma, resultados do próprio Censo das UBS sinalizam melhorias na infraestrutura entre 2012 e 2024, em todas as regiões brasileiras ^24^. O panorama mais favorável, provavelmente, é resultante de políticas convergentes para enfrentamento da escassez de profissionais, a exemplo do Programa Mais Médicos (PMM) ^25^, e de políticas mais recentes como o Informatiza UBS ^26^ e o programa SUS Digital ^27^, que buscam universalizar a informatização e diversificar as ferramentas de comunicação entre os serviços, os profissionais de saúde e a população. 

Os resultados ratificam a presença expressiva do prontuário eletrônico nas UBS. Nos últimos anos, houve importante crescimento e disseminação do Prontuário Eletrônico do Cidadão (PEC) e-SUS APS entre os municípios brasileiros, que, mesmo sem a obrigatoriedade de instalação e uso, passou a ser a principal escolha ^28^. Com vistas à coordenação do cuidado, o compartilhamento dos registros com algum outro ponto da RAS é sinalizado pela metade das UBS, com desigualdades regionais. Ainda que promissor, os resultados devem ser analisados com cautela. A maior parte do compartilhamento de informações clínicas via PEC ocorre entre UBS, enquanto com serviços fundamentais à continuidade assistencial - como CAPS, atenção especializada, urgências/UPA e CEO - permanece restrita. 

A ficha de referência/contrarreferência e as sessões clínicas conjuntas configuram as estratégias mais consolidadas. Embora as guias de referência e contrarreferência sejam os instrumentos há mais tempo disponíveis no SUS, as referências - da APS para outros serviços da RAS - são muito mais frequentes que o contrário, sinalizando uma comunicação de mão única. Estratégias de acomodação mútua, que envolvem contato direto entre profissionais de distintos serviços como as sessões clínicas compartilhadas, são consideradas essenciais para alcançar o cuidado colaborativo e estabelecer relações de confiança entre profissionais de saúde ^10^ ^,^ ^29^. Os resultados do Censo das UBS sinalizam maior utilização desta estratégia, se comparado a estudos realizados em outros contextos ^30^ ^,^ ^31^.

Instrumentos cruciais para condições complexas, como o PTS, o uso do telessaúde, consultas compartilhadas e o recebimento regular de resumo de alta hospitalar, apresentam baixa adesão. Conforme argumenta-se neste artigo, medidas pró-coordenação exigem ações e incentivos direcionados. Tomando o relatório de alta como exemplo, em um município brasileiro, sua institucionalização ocorreu por meio de um programa de residência médica em hospitais públicos, contribuindo para que o documento passasse a ser recebido com maior frequência na APS ^30^. Contudo, a condução do resumo de alta, da referência e contrarreferência pelo usuário parece ser o padrão predominante, comprometendo o recebimento e a chegada na APS em tempo oportuno para tomadas de decisão ^30^ ^,^ ^31^. 

Os resultados do Censo das UBS sinalizam importante frequência de compartilhamento do cuidado com os CAPS e hospitais. As reformas psiquiátrica e sanitária brasileiras compartilham princípios e propostas de mudanças no modelo de atenção à saúde. CAPS e UBS são serviços territoriais e, conforme observado por Miliauskas et al. ^32^, parece haver uma associação entre aumento simultâneo da oferta desses serviços e a redução das taxas de internações psiquiátricas em regiões metropolitanas. Esse achado pode sinalizar que, além da ampliação da cobertura, podem estar em curso estratégias compartilhadas de cuidados.

A presença de gerência na UBS aparece como fator-chave mesmo em contextos mais vulneráveis: unidades com gerência apresentam maior uso combinado do aplicativo WhatsApp, telefone e e-mail e maior frequência de comunicação e adoção de mecanismos de coordenação clínica. Os achados reforçam que o planejamento e a organização dos processos de trabalho na APS são determinantes para viabilizar e qualificar a troca de informações com outros níveis assistenciais, sinalizando que o fortalecimento dessa função - com profissionais com formação na área de gestão em saúde, capacidade para análise e utilização de indicadores, trabalho em equipe e comunicação, entre outras habilidades e competências ^33^ ^,^ ^34^ - pode atenuar desigualdades territoriais. A ausência de formação específica para o desenvolvimento da gerência e o acúmulo de funções gerenciais e assistenciais, principalmente em UBS com maior número de equipes, são fatores críticos que podem comprometer a atuação destes profissionais, sobretudo em atividades que demandam articulação com os demais serviços da RAS ^34^. Embora o artigo não apresente análises relacionadas à formação da gerência das UBS (variável presente no instrumento do Censo das UBS), os achados sinalizam que o perfil profissional delineado ao longo da discussão converge com as competências presentes na formação do sanitarista, particularmente do bacharel em Saúde Coletiva.

Nesse viés, as desigualdades entre áreas urbanas e rurais são fatores críticos. Em municípios urbanos, o contato telefônico e via e-mail é expressivamente maior que em áreas rurais. Essa diferença se reproduz no compartilhamento do cuidado na RAS: UBS localizadas em municípios urbanos apresentam maior articulação com CAPS, hospitais, maternidades, serviços especializados e de IST, enquanto áreas rurais enfrentam maiores dificuldades de integração com serviços de saúde mental, saúde bucal especializada e reabilitação - reflexos, também, da dificuldade de acesso à retaguarda terapêutica nessas localidades ^35^.

Os encaminhamentos via sistema de regulação ocorrem em cerca de dois terços das UBS, paralelamente a outros fluxos sob responsabilidade do próprio usuário. Estabelecer os serviços de APS com função de *gatekeeper* não é suficiente para a garantia de coordenação ou acesso. Para tanto, é necessário assegurar uma oferta pública suficiente de serviços, organizar as redes de cuidado e potencializar a capacidade resolutiva na APS, com funções de coordenação estabelecidas, para que os pacientes não precisem realizar percursos circulares, decorrentes de barreiras burocráticas no processo de regulação assistencial ^36^. A prática comum de entregar uma ficha de encaminhamento física desresponsabiliza a UBS pela garantia da continuidade do cuidado e abre espaço para a busca de serviços no setor privado, com ampliação do uso de “planos populares”, amplificando o entrelaçamento público-privado ^37^. Ao mesmo tempo, a inserção da solicitação no sistema de regulação não pode ser um ato burocrático assumido como finalização da responsabilidade da UBS ^38^, o que parece ser frequente à medida que somente cerca de um terço mantém listas de pacientes encaminhados ou monitoram o tempo de espera.

A aceitação de encaminhamentos para especialidades realizados por enfermeiras em mais da metade das UBS denota a importância crescente destes profissionais na APS. A maior prevalência de encaminhamentos realizados por enfermeiras no Nordeste pode estar relacionada ao contexto histórico, com universalização mais precoce de cobertura pela ESF, à atuação no programa de agentes comunitários de saúde (ACS) ^39^, e à vacância de profissionais médicos nas equipes, prévia à implantação do PMM ^24^. Na mesma direção, é cada vez mais frequente a implantação de protocolos abrangentes para a atuação desses profissionais e para a presença de residência em enfermagem de família e comunidade nas UBS, principalmente nos centros urbanos ^40^. 

As associações observadas entre a maior prevalência de estratégias de regulação e a presença de gerência, de pelo menos um médico especialista em MFC - com maior número de eSF na UBS - sinalizam a necessidade de ampliação da infraestrutura dos serviços e do corpo técnico. Nesse sentido, as ações de coordenação compreendem funções gerenciais ^41^, o que implica em profissionais com tempo protegido para o exercício destas funções, na estruturação das UBS para qualificar encaminhamentos e no monitoramento da continuidade dos cuidados. Ademais, médicos com formação na especialidade MFC bem integrados em equipes multiprofissionais são reconhecidos como fundamentais para a qualidade, coordenação do cuidado e abordagem de uma APS integral ^42^ ^,^ ^43^. 

Um dos limites deste estudo refere-se à natureza transversal do Censo das UBS, que não possibilita estabelecer relações de causalidade. Além disso, as informações foram obtidas por autodeclaração dos respondentes, o que pode implicar um viés informacional. Desse modo, não é possível inferir a qualidade, intensidade e o uso rotineiro dos mecanismos de coordenação, assim como possíveis repercussões nas trajetórias assistenciais dos usuários, sinalizando necessidade de estudos complementares.

## Considerações finais 

Os resultados evidenciam que, embora tenham sido identificados avanços importantes, a coordenação do cuidado a partir da APS no Brasil permanece fortemente condicionada por fragilidades estruturais, desigualdades territoriais e pela predominância de mecanismos informais de comunicação. As desigualdades regionais, urbano-rurais e organizacionais - especialmente associadas à infraestrutura, disponibilidade de profissionais e presença de gerência nas UBS - reforçam que o desempenho da coordenação é dependente de capacidades institucionais, organizacionais e gerenciais concretas, operadas no nível local.

Para superar as barreiras identificadas, recomenda-se o fortalecimento de políticas estruturantes que qualifiquem a APS como coordenadora do cuidado, com investimentos contínuos em infraestrutura digital, conectividade de qualidade e dispositivos móveis institucionais com planos de dados. É fundamental avançar na interoperabilidade dos prontuários eletrônicos, fluxos de comunicação e interação interprofissional na RAS, reduzindo a responsabilização do usuário pela busca dos serviços e do trânsito da informação clínica. Ademais, destaca-se a necessidade de fortalecer a função gerencial nas UBS, com profissionais com formação em gestão em saúde, tempo protegido, educação permanente e suporte técnico para o planejamento, monitoramento e articulação da rede. Por fim, ressalta-se a necessidade de ampliar a capacidade resolutiva da APS por meio do trabalho em equipe multiprofissional, valorizando o papel ampliado da enfermagem, da MFC, bem como da contribuição de outras categorias estratégicas - a exemplo dos sanitaristas e demais profissionais que compõem as equipes multiprofissionais (eMulti) na APS.

## Data Availability

As fontes de informação utilizadas no estudo estão indicadas no corpo do artigo.
